# A real-time detection method for classification and grading of multi-variety shiitake mushrooms in natural growth environments

**DOI:** 10.3389/fpls.2026.1919271

**Published:** 2026-08-26

**Authors:** Xinfa Wang, Yongqi Zhu, Huamei Cao, Xuan Wen, Songyi Wang, Yafeng Han, Yuxin Tong, Xiujuan Wan, Bihua Chen, Chaohui Zhang, Guang Zhang

**Affiliations:** 1 School of Computer Science and Technology, Henan Institute of Science and Technology, Xinxiang, China; 2 Xinxiang City Key Laboratory of Intelligent Plant Factory, Xinxiang, China; 3 Institute of Environment and Sustainable Development in Agriculture, Chinese Academy of Agricultural Sciences, Beijing, China; 4 Zhengzhou Academy of Agricultural Science and Technology, Zhengzhou, China; 5 School of Horticulture and Landscape Architecture, Henan Institute of Science and Technology, Xinxiang, China; 6 School of Life Sciences, Henan Institute of Science and Technology, Xinxiang, China

**Keywords:** classification and grading, deep learning, precision agriculture, production estimate, shiitake mushroom

## Abstract

The intelligent production of edible fungi requires accurate and real-time monitoring of mushroom growth status throughout cultivation. However, real-time classification and grading of multiple shiitake mushroom varieties in natural environments remain challenging due to illumination variation, pose diversity, occlusion, dense small targets, and subtle inter-variety morphological differences. To address these task-specific challenges, this study proposes a lightweight real-time detection framework for fine-grained shiitake mushroom recognition. The model is designed from a problem-driven perspective by jointly considering deployment efficiency, small-object perception, and subtle feature discrimination. First, a Spatially-weighted Token Gated Convolution (STGConv) module is proposed to achieve lightweight feature extraction by reducing computational redundancy while maintaining robust feature learning. Second, an Adaptive Residual Fusion Head (ARFHead) is proposed to enhance multi-scale feature interactions and improve the perception of densely distributed, partially occluded mushrooms. Finally, a Dynamic Sampling operator is incorporated into the upsampling process to adaptively restore critical detail features and improve discrimination of subtle differences among varieties and growth grades. This modular combination provides a balance between lightweight computation, small-object perception, and fine-detail reconstruction, making it suitable for agricultural vision tasks in natural growth environments. Experimental results show that the proposed model achieves an mAP@0.5 of 97.9%, precision of 96.0%, recall of 96.3%, and 110.8 FPS, with a model size of only 8.27 MB, indicating its suitability for real-time monitoring and edge deployment in practical cultivation environments.

## 1 Introduction

Shiitake mushrooms are nutrient-rich edible fungi with a tender texture and distinctive flavor. They possess high culinary value and offer medicinal and health benefits, making them indispensable in people’s daily diets and healthy lifestyles (Shi et al., 2024). Market demand has steadily increased in many countries, with production scales expanding (Vaishnavi et al., 2022). However, production across all stages relies heavily on manual and semi-automated machinery, resulting in low efficiency, high wastage, and elevated labor costs. Improvements in output and quality have lagged behind market demands, making it imperative to enhance mechanization, automation, and intelligent application in shiitake mushroom production (Xu and Zhang, 2021). Monitoring their growth status, developmental stage, and quality development is fundamental for intelligent shiitake mushroom cultivation. Under natural growth conditions, shiitake mushrooms are primarily cultivated using mushroom logs as substrates, with multiple mushrooms capable of growing simultaneously on each log. These mushrooms exhibit significant variations in size, shape, maturity, and visual integrity. Different shiitake varieties also demonstrate diversity in morphological characteristics and growth rhythms (Wang and Zhao, 2023; Cheute et al., 2022). Shiitake mushrooms’ disordered growth and dense distribution pose significant challenges for rapid, real-time, and precise detection of their growth status, quality identification, and grading classification. Given the terminal devices’ limited computational and storage capabilities, these tasks remain largely unresolved and have yet to be fully implemented. Therefore, attaining automated identification and grading of shiitake mushrooms in their natural state would facilitate accurate monitoring of growth dynamics and maturity trends across different varieties and provide robust support for developing intelligent harvesting technologies. This advancement would effectively elevate the level of automation and mechanization in shiitake mushroom production.

Computer vision technology has recently been widely applied in agricultural inspection, demonstrating significant research value and application prospects (Wang et al., 2025). The rapid advancement of deep learning technologies has enabled neural network-based object detection algorithms to identify and classify agricultural products efficiently (Wang et al., 2023). Object detection algorithms primarily fall into two categories: one is the two-stage approach based on detection frames and classifiers (Zhao et al., 2025). Like the R-CNN (Wang and Liu, 2021) series of algorithms, it offers high accuracy but operates slowly. Related research includes (Yang et al., 2018) explored the application of computer vision in livestock monitoring, employing Faster R-CNN to recognize individual pig behaviors and developing image-based algorithms to assess feeding and health status (Wismadi et al., 2020). explored a compact VGGNet-inspired architecture for assessing the harvest readiness of dragon fruit. By employing a convolutional neural network (CNN), the fruits were classified into two categories: ready for harvest or still unripe (Kang and Chen, 2020). developed a deep learning-based framework for real-time fruit detection in apple orchards, enabling efficient apple-picking (Yu et al., 2019). developed a real-time strawberry detection method for unstructured environments by using ResNet-50 (He et al., 2016) with a Feature Pyramid Network (FPN) (Lin et al., 2017) as the backbone and integrating it with Mask R-CNN (He et al., 2017). Similarly (Mu et al., 2020), employed Faster R-CNN combined with ResNet-101 and applied transfer learning to 60 identify immature tomatoes.

Another approach is based on regression-based first-order algorithms, such as the YOLO (Zheng et al., 2022) series, which offer faster inference speeds and greater practicality (Yao et al., 2021). The YOLO series of object detection algorithms has become a significant research focus in agricultural visual inspection due to its highly efficient real-time detection capabilities (Jin et al., 2025; Cemalettin Akdoğan et al., 2025) (Lin et al., 2025). proposed an improved algorithm, DPD-YOLO, which combines BiFPN, a small object detection layer, a coordinate attention mechanism, and an RT-DETR detection head, significantly enhancing detection capabilities for densely clustered pineapples (Yang et al., 2025). introduced an adaptive weighted knowledge distillation strategy in YOLOv7-tiny, effectively improving the accuracy of strawberry growth state detection (Chen et al., 2023). proposed a lightweight multi-class occluded object detection method for camellia seeds called YOLO-COF. Within the YOLOv5s framework, they introduced the K-means++ clustering algorithm to filter and select target datasets automatically. The successful application of YOLO models in fruit and vegetable detection offers valuable insights for edible mushroom detection. Increasing research on shiitake mushroom detection, especially in cultivation and management, stems from their high visual similarity, indistinct maturity levels, and frequent exposure to complex environments (Liu et al., 2022). proposed an efficient channel pruning mechanism to enhance the YOLOX deep learning approach, enabling effective detection of shiitake mushroom surface textures for quality identification and grading (Lu and Liaw, 2020). proposed a mushroom image measurement algorithm based on convolutional neural networks to calculate mushroom cap diameters. This algorithm is used for real-time image measurement and contour extraction during mushroom growth (Javanmardi and Ashtiani, 2025). proposed a deep learning-based framework for predicting mushroom shelf life. This framework non-destructively assesses the freshness of different mushroom varieties under refrigeration conditions, achieving high-precision classification of storage time (Tao et al., 2024). proposed ReYOLO-MSM, an improved YOLO-based method that integrates monocular vision and stick–mushroom growth relationships, using rotating elliptical bounding boxes to improve detection accuracy and enable mushroom counting, size and roundness measurement, and harvesting status assessment.

Existing studies related to mushroom analysis mainly focus on specific and isolated tasks, such as species identification, quality assessment, or single-stage detection under controlled conditions. However, practical shiitake mushroom production environments involve multiple varieties, complex backgrounds, illumination variations, and frequent occlusion, making it challenging for existing methods to achieve accurate and real-time classification and grading simultaneously. Therefore, there remains a research gap in developing lightweight and robust frameworks capable of multi-variety recognition and growth-stage assessment under natural cultivation conditions. In actual mushroom production, mixed cultivation of multiple shiitake varieties is common. Traditional methods typically rely on manual identification or hanging labels before the racks for differentiation. This approach is cumbersome, prone to errors, and challenging in the complex mushroom shed environment. The diverse morphology of shiitake mushrooms, dense growth patterns, severe shading, and uneven lighting conditions make detection extremely difficult, severely compromising the accuracy and robustness of identification (Peng et al., 2023). Based on the issues above, this study focuses on the detection task of multiple fresh shiitake mushroom varieties in mushroom sheds under natural growth conditions. Through model selection experiments, we propose YOLO-RTSCGD (Real-Time Shiitake Classification and Grading Detection), an efficient detection model for shiitake growth status, built on YOLO11n as the baseline. This model enhances detection speed by optimizing the network architecture and feature extraction mechanism while effectively improving recognition accuracy for multiple varieties of shiitake mushrooms and their different growth stages. The model can monitor shiitake mushroom growth in real time, assist in analyzing growth trends, determine the best time to pick, predict the next harvesting cycle, and timely identify and process deformed mushrooms, thereby improving the intelligence level of shiitake mushroom cultivation, the marketability of finished products, and overall output. It also lays the foundation for intelligent perception in complex environments for fruit and vegetable harvesting 108 robots (Saleem et al., 2021; Fang et al., 2021; Zulkifley et al., 2022).

The main contributions of this work are summarized as follows.

- We constructed a dataset of multi-variety and multi-grade shiitake mushroom images under natural growth conditions and designed a multi-dimensional labeling system that integrates variety and quality grade. We divided shiitake mushroom targets into 12 categories and adopted structured coding rules to enhance category discrimination and label semantic expression capabilities.
- We propose an improved YOLO-RTSCGD shiitake mushroom detection model that maintains lightweight characteristics while significantly enhancing detection accuracy and recognition performance for small and occluded targets.
- We used the PySide6 framework and OpenCV library to integrate the lightweight model into a Raspberry Pi, enabling real-time detection and analysis of shiitake mushroom growth status on a computer, and independent counting of shiitake mushrooms of different varieties and grades in the image. In addition, we developed an application using the NCNN framework and successfully performed real-time and fast shiitake mushroom detection on an Android terminal.

## 2 Materials and methods

### 2.1 Data collection

**Figure 1:**
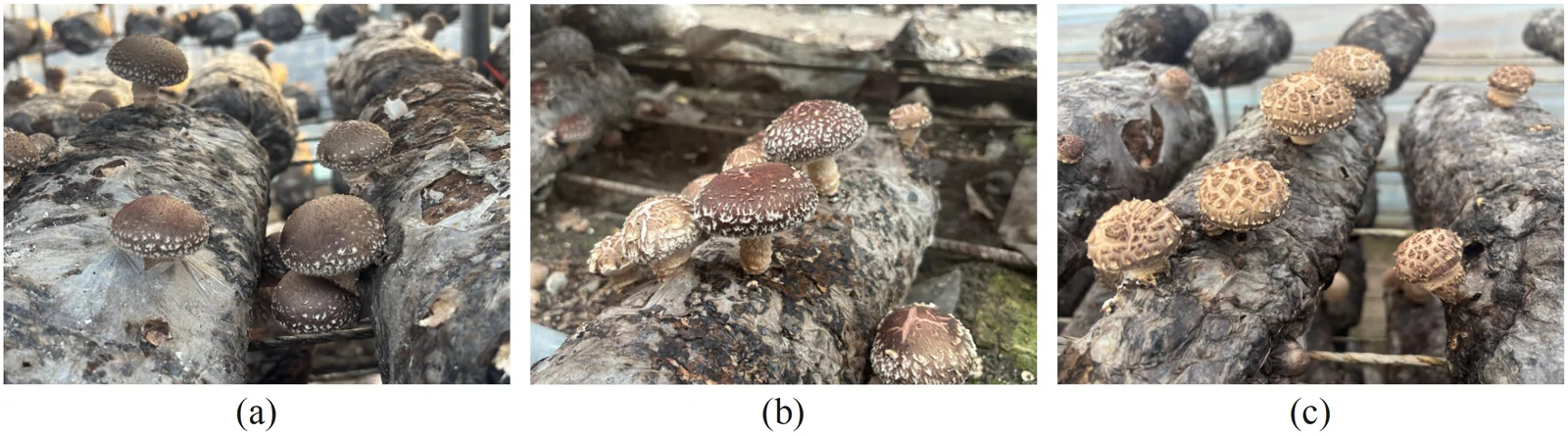
Original images of three shiitake mushroom varieties: **(a)** 9608; **(b)** Chunsheng No. 1; **(c)** Qihe No. 9.

All shiitake mushroom image data used in this study were collected from edible fungus production bases served by our research team. These include five production sites such as Beiwudu Town, Wuyang County, Luohe City, Henan Province, China (33^◦^37^′^23.27^′′^N, 113^◦^39′25.88^′′^E) and three shiitake mushroom cultivation enterprises, including Yufu Mushroom Industry(35^◦^16′45.11^′′^N, 113^◦^43′59.44^′′^E) in Huojia County, Xinxiang City, Henan Province, China. The dataset comprises 3,150 raw image files accumulated over nearly three years of continuous work. The image acquisition equipment included SONY A6400, iPhone 15 Plus, Vivo NEX, and other mobile devices. The image resolutions include six specifications, such as 2016×1512 pixels, 2312×1736 pixels, 2856×2142 pixels, etc. Moreover, continuously adjust shooting parameters such as angle, position, light intensity, aperture, and shooting distance during the shoot. These datasets encompass naturally occurring shiitake mushroom instances across multiple geographical regions, light conditions, clarity levels, angles, scales, and fields of view. The extensive and reliable data collection and comprehensive annotation ensure the model’s adaptability and generalization in complex environments. It also enhances robustness and accuracy in real-world production scenarios. This study primarily selected three commercially available shiitake mushroom varieties, 9608, Chunsheng No. 1, and Qihe No. 9, as experimental subjects. These three varieties exhibit high similarity in appearance, including shape, color, and cap pattern, making them challenging to distinguish for non-specialists. This selection provides strong relevance and challenge for multi-variety shiitake classification and grading testing. Images of three varieties of shiitake mushrooms are shown in **Figure 1**.

Compared with existing public datasets and datasets used in related studies, the proposed shiitake mushroom dataset exhibits several distinctive characteristics. Most existing datasets are collected under controlled conditions or focus on post-harvest analysis. In contrast, the proposed dataset is collected directly from natural cultivation environments, featuring complex backgrounds, variable illumination, dense spatial distribution, and frequent occlusion. Moreover, the dataset covers multiple shiitake varieties and explicitly annotates different growth grades, enabling simultaneous multi-variety classification and grading within a single detection framework. These characteristics make the dataset particularly suitable for evaluating real-time detection algorithms under practical production conditions.

**Figure 2:**
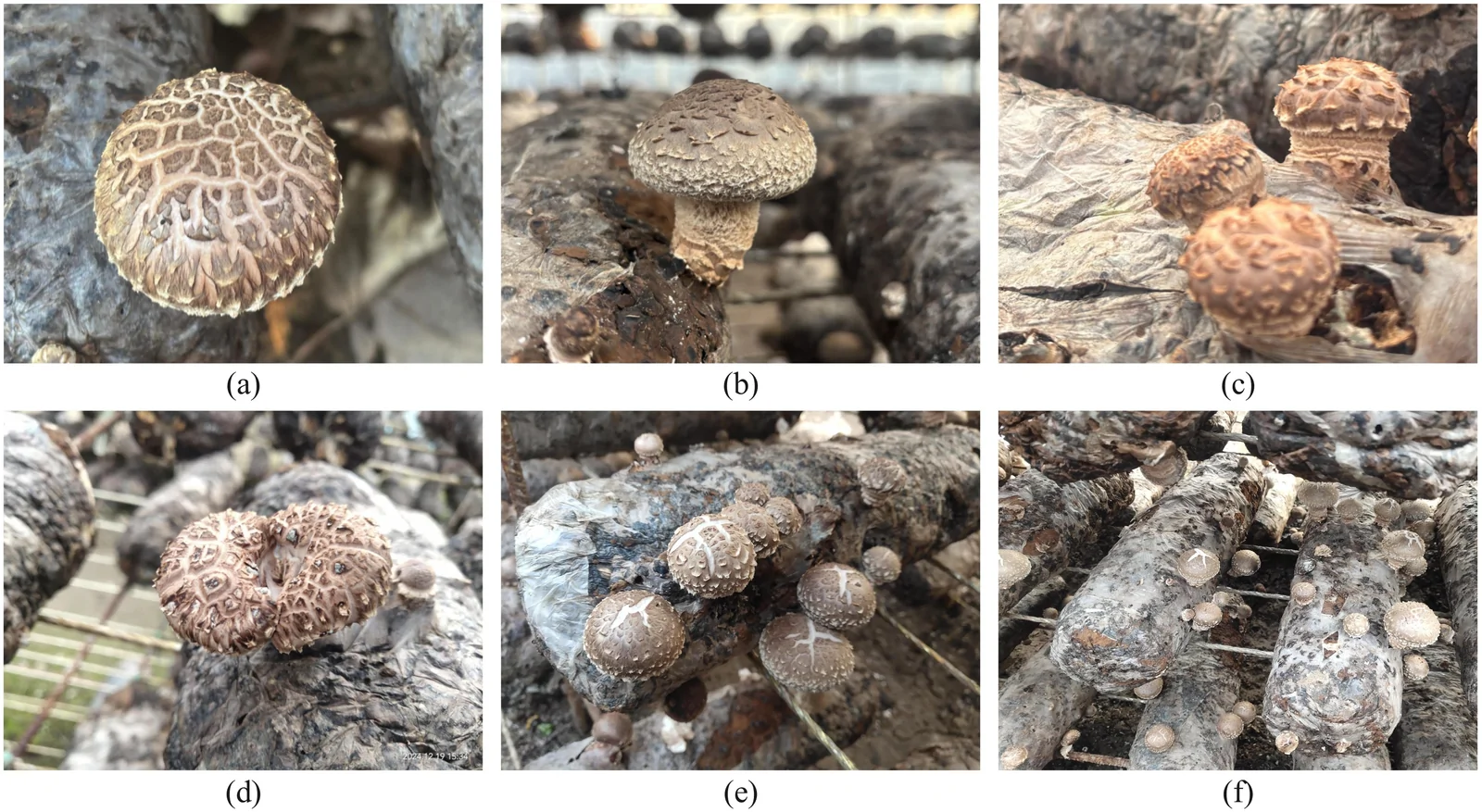
Morphological characteristics of different categories of shiitake mushrooms in natural growth images: **(a)** Mature shiitake mushrooms; **(b)** Growing shiitake mushrooms; **(c)** Juvenile shiitake mushrooms; **(d)** Deformed shiitake mushrooms; **(e)** Densely overlapping; **(f)** Wide-field view.

Following international standards and common market classification methods in China, while considering practical application and research suitability, shiitake mushrooms in each growth stage are categorized into four grades: mature-stage shiitake, growing-stage shiitake, juvenile-stage shiitake, and deformed shiitake (Yin et al., 2022). Mature shiitake mushrooms exhibit a larger appearance, with caps partially open to 60-90% and edges slightly curled inward. Growing-stage shiitake mushrooms exhibit a medium size, with caps opened to 60% or less, or not yet opened. Juvenile shiitake mushrooms appear small, having just begun growing on the substrate. Deformed shiitake mushrooms show significant color variation on the surface and typically have irregular caps. The shapes and forms of shiitake mushrooms in different categories, as observed in natural growth images, are shown in **Figure 2**.

### 2.2 Data preprocessing and annotation

All collected image data were systematically screened before labeling the shiitake mushroom image data. First, images lacking variety labels at acquisition and those that could not be reliably identified were removed. Second, necessary cropping and format conversion were performed on specific images to ensure consistency in data formats. Finally, key attributes of the image data were batch-modified, specifically clearing the “EXIF” attributes, to ensure that the annotated data used for model training remained consistent with the original annotations.

**Figure 3:**
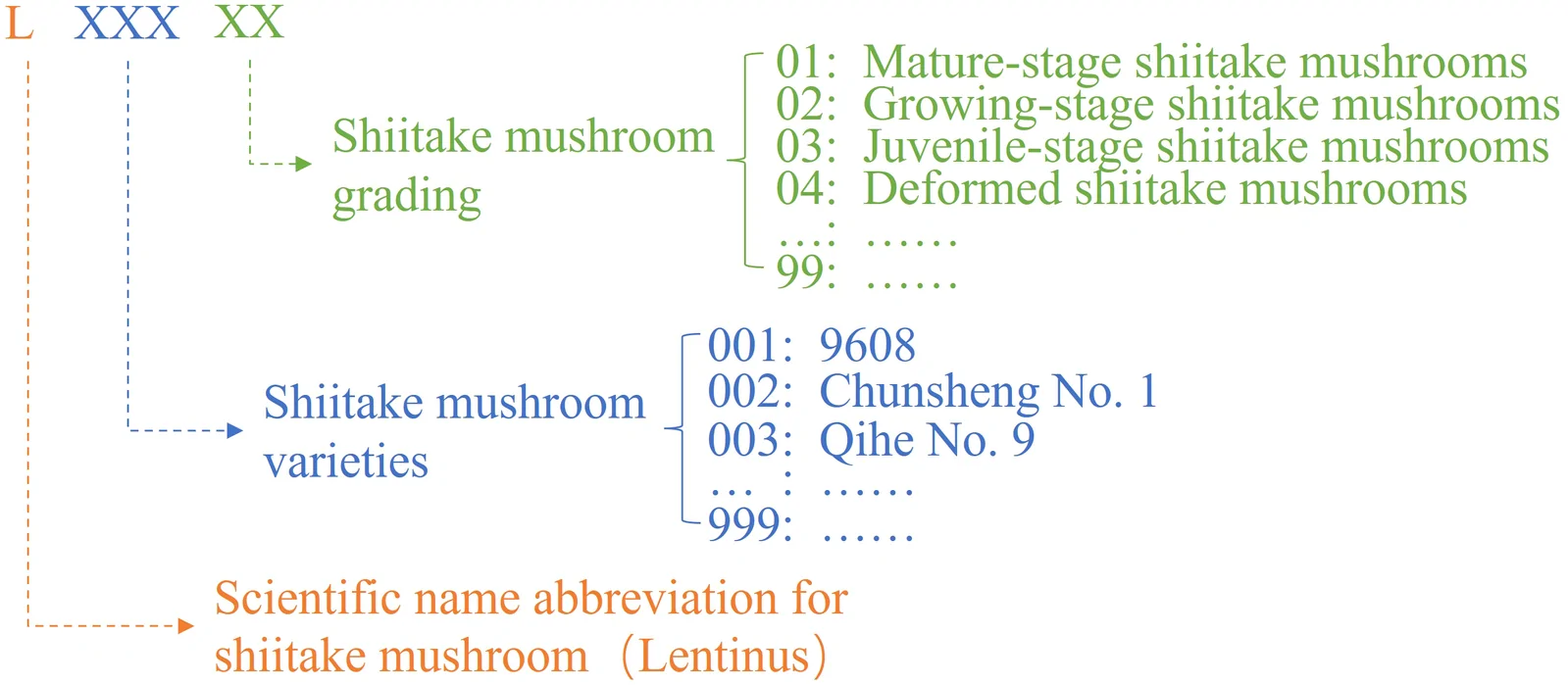
Structured tag naming conventions.

This study employed LabelImg, a widely used data annotation tool, to classify and annotate mushroom instances in images of naturally grown shiitake mushrooms. The specific annotation method was: (1) Conduct a comprehensive manual evaluation of each shiitake mushroom instance in the image to determine its category and grade. (2) All mushroom instances in the image are accurately marked with tight bounding boxes and named with corresponding labels. (3) After labeling all manually identifiable shiitake mushroom instances in an image, save the corresponding.txt label file. (4) Proceed to annotate the shiitake mushroom example in the following image. A full-instance manual annotation strategy was adopted to ensure the comprehensiveness and accuracy of data annotation. All visible individual shiitake mushrooms in the images were manually identified and labeled, including partially obscured mushrooms. For these cases, reasonable inferences were made based on the morphological characteristics of the visible portions to provide complete annotations for the hidden parts. Regarding label naming, we proposed a simplified multidimensional label nomenclature specification, which makes the annotated data concise and accurate, ensuring the standardization of the dataset, as shown in **Figure 3**:

Taking the 9608 variety as an example, mature shiitake mushrooms are labeled L00101, growing-stage shiitake mushrooms are L00102, juvenile-stage shiitake mushrooms are L00103, and deformed shiitake mushrooms are L00104. Here, L denotes shiitake mushroom, 001 represents the variety code, and the last two digits indicate the growth stage category. The other two varieties follow the same naming convention: in the Chunseng No. 1 variety, the four shiitake mushroom types are labeled L00201-L00204; in the Qihe No. 9 variety, the four shiitake mushroom types are labeled L00301-L00304. A shiitake mushroom detection dataset comprised three varieties, four categories, and 12 labels. Data annotation was completed by members of the research team under the guidance of edible edible fungi experts. We conducted small-batch annotation, preliminary model training, and trial inference testing before large-scale data annotation. Subsequently, the dataset annotation process was conducted in two rounds. In the first round, trained annotators performed the initial annotation of mushroom instances according to predefined annotation guidelines. In the second round, the annotated results were carefully reviewed by other team members and edible fungi experts, and inconsistent labels, inaccurate bounding boxes, and potential annotation errors were corrected.

### 2.3 Data augmentation

**Figure 4:**
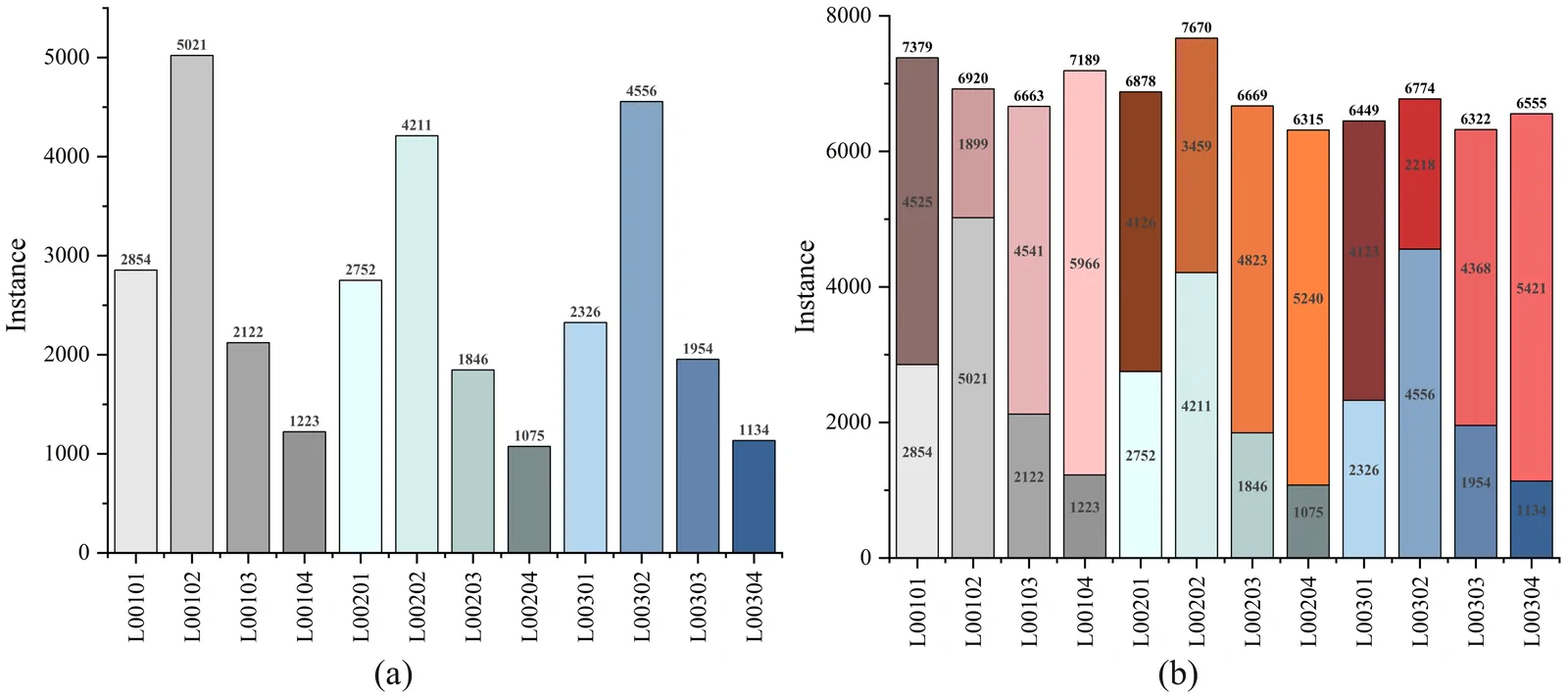
Instance Distribution Diagram: **(a)** Instance distribution diagram before data augmentation; **(b)** Instance distribution diagram after data augmentation.

The complete distribution of annotations in the dataset directly impacts the accuracy and effectiveness of model training. Therefore, we first analyzed the distribution of annotated instances across different shiitake mushroom categories. The statistical results and instance distribution are shown in **Figure 4**. The statistical results indicate that the dataset contains 30,355 annotated shiitake mushroom instances, with significant differences in sample sizes across different categories. Most specimens are in the growth stage, accounting for 45% of the total. Those in the mature and juvenile stages comprise 26% and 20% respectively, with relatively balanced distributions. Deformed specimens are comparatively scarce, constituting less than 10%. This pattern is consistent with practical shiitake mushroom cultivation practices. The distribution of instances across the three shiitake mushroom varieties used in the experiment accounted for 37%, 32%, and 31% respectively, with the labeled instance counts distributed relatively evenly.

Data augmentation is an essential step in training object detection models. In this study, a random combination strategy of multiple augmentation techniques was adopted to enrich and expand the original dataset, compensate for the scarcity and limitations of the collected samples, alleviate sample imbalance, and improve the model’s ability to detect mushroom images under complex environments. Data augmentation generates new image samples by applying random rotation, cutout, brightness adjustment, and noise injection to the original images. The augmentation process can be formulated as shown in Equation 1:

(1) $$X_{\text{aug}}=T\left(X_{i}\right)$$

where *X_i_* represents the original image, *X*_aug_ represents the augmented image, and *T*(·) denotes the randomly selected combination of augmentation operations.

**Figure 5:**
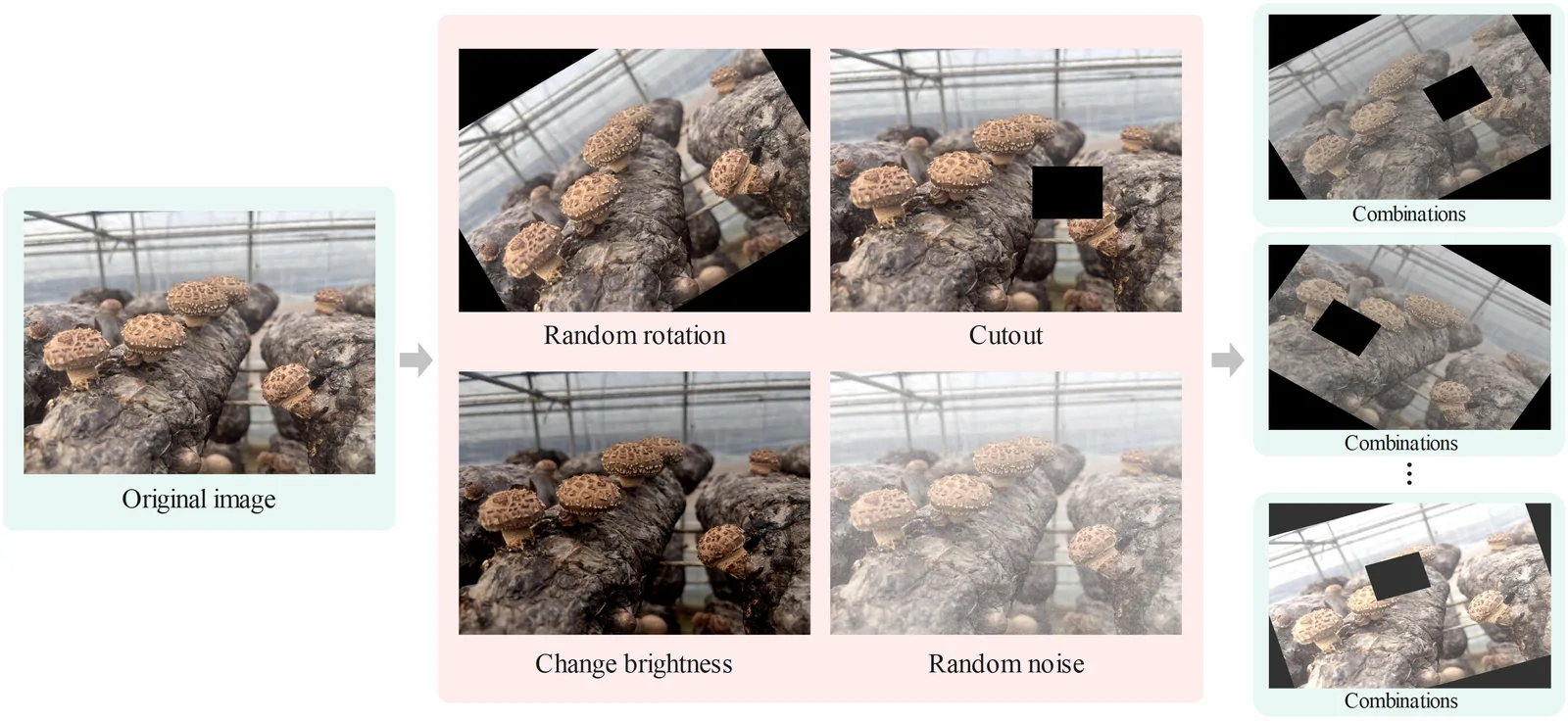
Various types of data augmentation effects.

Random rotation simulated variations from different shooting angles and orientations, enhancing the model’s robustness to spatial transformations. Random adjustment of image brightness enhances the model’s adaptability to lighting variations, compensating for the complex and variable lighting conditions within shiitake mushroom cultivation greenhouses during original image data acquisition. Random noise improves the model’s robustness to potential sensor or environmental disturbances during image acquisition. The cutout method randomly obscures partial regions of an image to enhance the model’s recognition capability when target parts are missing or occluded. Randomly combining the four data augmentation methods can significantly enrich and expand the dataset. The data augmentation patterns and effects are shown in **Figure 5**.

An innovative approach was employed and systematically evaluated during the data augmentation process. Instead of applying proportional and uniform data augmentation to the original images, a differentiated augmentation strategy was adopted based on the distribution of instances across different categories. Multiple data augmentation methods are applied for categories with fewer samples to expand the sample size, while categories with larger sample sizes receive fewer augmentation techniques. This approach ensures that the distribution of samples in the augmented dataset is broadly balanced. The distribution of samples in the augmented dataset is shown in **Figure 4**. The enhanced image dataset has expanded to 8,432 images, with potential for further growth. The total number of annotated shiitake mushroom instances in the enhanced dataset reached 81,783. This approach improves dataset completeness and compensates for the imbalance of shiitake mushroom instances in images collected from actual cultivation environments.

### 2.4 Baseline model selection

This study primarily employs intelligent vision technology to design a deep learning model tailored for shiitake mushroom image detection. The model addresses the classification and grading tasks of diverse shiitake varieties, aiming to enhance the advancement of intelligent applications in shiitake cultivation. The model must adapt to the varied poses, occlusions, and lighting variations encountered in natural growth conditions while supporting deployment on both desktop and mobile platforms. Desktop deployment leverages computers’ computational power and storage advantages, prioritizing precision and stability. Mobile deployment emphasizes a lightweight model design and real-time capabilities to accommodate mobile smart devices’ limited storage and computational resources (Wang et al., 2022). Current mainstream detectors such as Faster R-CNN and SSD generally require substantial computational resources and have relatively slow inference, which limits their practicality on edge devices (Wu et al., 2024). In contrast, the YOLO family of models offers a more favorable trade-off between accuracy and speed, with YOLO11 excelling in structural optimization, feature extraction efficiency, and multi-scale perception. Considering model size, detection speed, accuracy performance, and adaptability to multi-scale targets, the relatively lightweight YOLO11n was selected as the baseline model.

This model features a compact size, fast inference speed, and low resource consumption, making it suitable for real-time detection on mobile devices and edge computing systems. It also exhibits strong performance in detecting objects of various types and scales. However, YOLO11n still has limitations in distinguishing similar varieties, detecting small objects, and maintaining robustness under complex lighting conditions. It inadequately extracts features from subtle morphological variations in shiitake mushrooms and is susceptible to background interference, reducing detection accuracy. Therefore, this study performs structural optimization and feature enhancement based on YOLO11n to improve the model’s accuracy, stability, and adaptability to complex environments.

### 2.5 Improved YOLO-RTSCGD model

Depending on the specific detection targets and application scenarios, detection methods may exhibit distinctive characteristics, and their algorithmic models and structures may also differ. Therefore, specialized research and validation of algorithms and models are required. This study primarily addresses accurately identifying shiitake mushroom varieties with highly similar morphological characteristics, improving classification accuracy and discriminative capability across different growth stages in complex environments. It proposes the YOLO-RTSCGD model for the classification and grading detection of naturally grown shiitake mushrooms. This model adopts a hierarchical architecture based on an encoder-decoder structure composed of the Backbone, the Neck, and the Detection Head. The overall architecture follows the feature pyramid paradigm of modern convolutional neural networks, achieving efficient object detection through multi-scale feature extraction and fusion.

**Figure 6:**
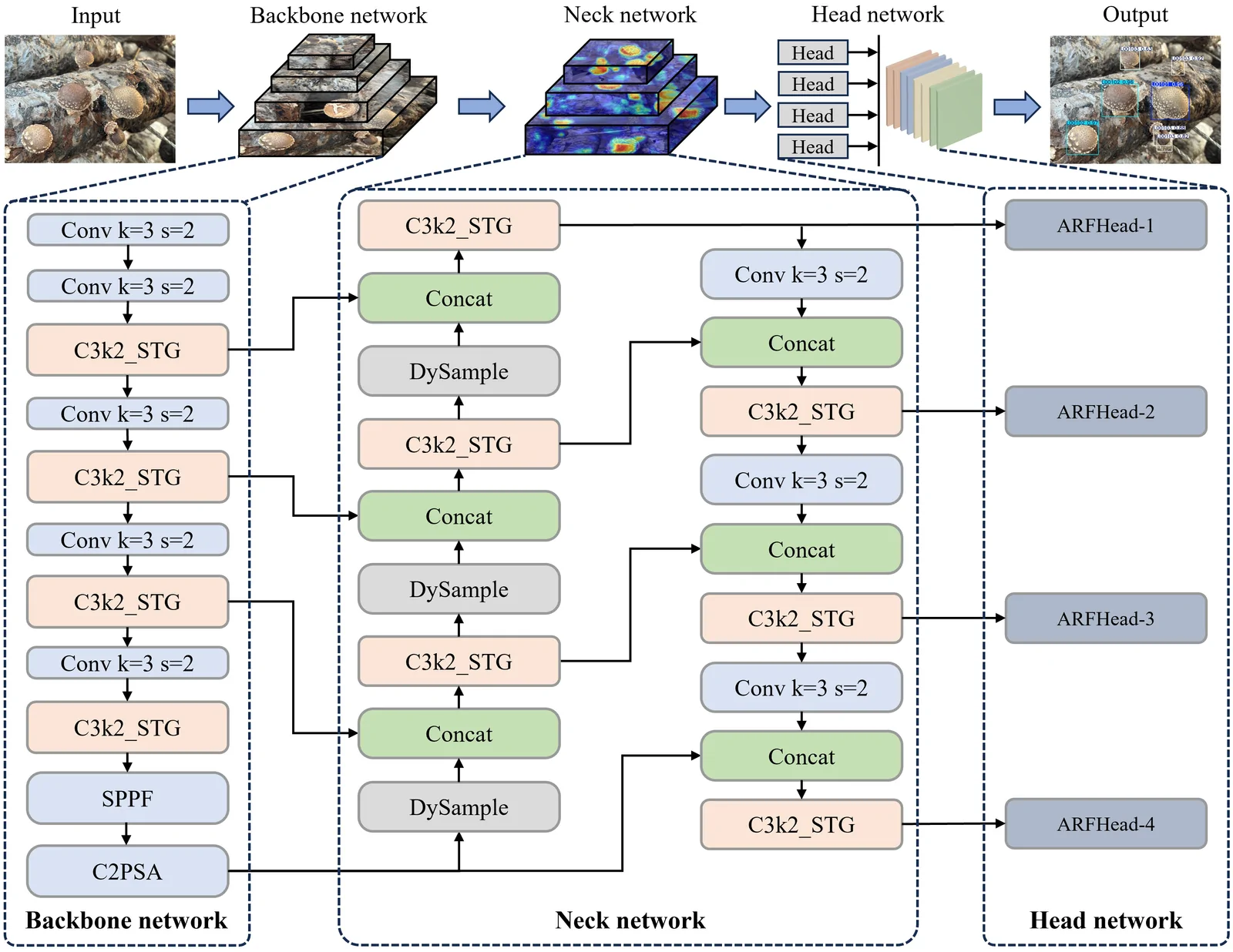
YOLO-RTSCGD model architecture.

The Backbone serves as the feature-extraction network and employs a hierarchical downsampling strategy to progressively encode input images. Two initial 3×3 convolutions with a stride of 2 perform early spatial compression, followed by multiple stages comprising C3k2 STG blocks and stride-2 convolutions that gradually enlarge channel capacity while constructing multi-scale feature representations. The Spatially-weighted Token Gated Convolution (STGConv) embedded in C3k2 STG reduces computational redundancy while preserving discriminative spatial information. In the final stage, the SPPF module enlarges the receptive field through multi-scale pooling, and the C2PSA module further enhances feature responses, producing semantically rich representations for subsequent detection. The Neck adopts a bidirectional feature aggregation architecture that combines top-down and bottom-up information propagation. Multi-scale features are progressively fused via feature concatenation and C3k2 STG refinement, while DySample is used during upsampling to adaptively restore fine-grained spatial details. This design effectively integrates shallow texture information with deep semantic representations, thereby improving the perception capability for densely distributed, partially occluded, and scale-varying shiitake mushrooms. The detection stage employs four Adaptive Residual Fusion Heads, each corresponding to a feature map at a different resolution. By aggregating residual features and interacting across scales, the proposed head enhances feature complementarity across multiple scales. It enables accurate localization and classification of mushrooms with diverse sizes and growth stages. The overall architecture effectively balances detection accuracy and computational efficiency, making it suitable for real-time deployment in practical cultivation environments. The complete network architecture is illustrated in **Figure 6**.

#### 2.5.1 Spatially-weighted token gated convolution

**Figure 7:**
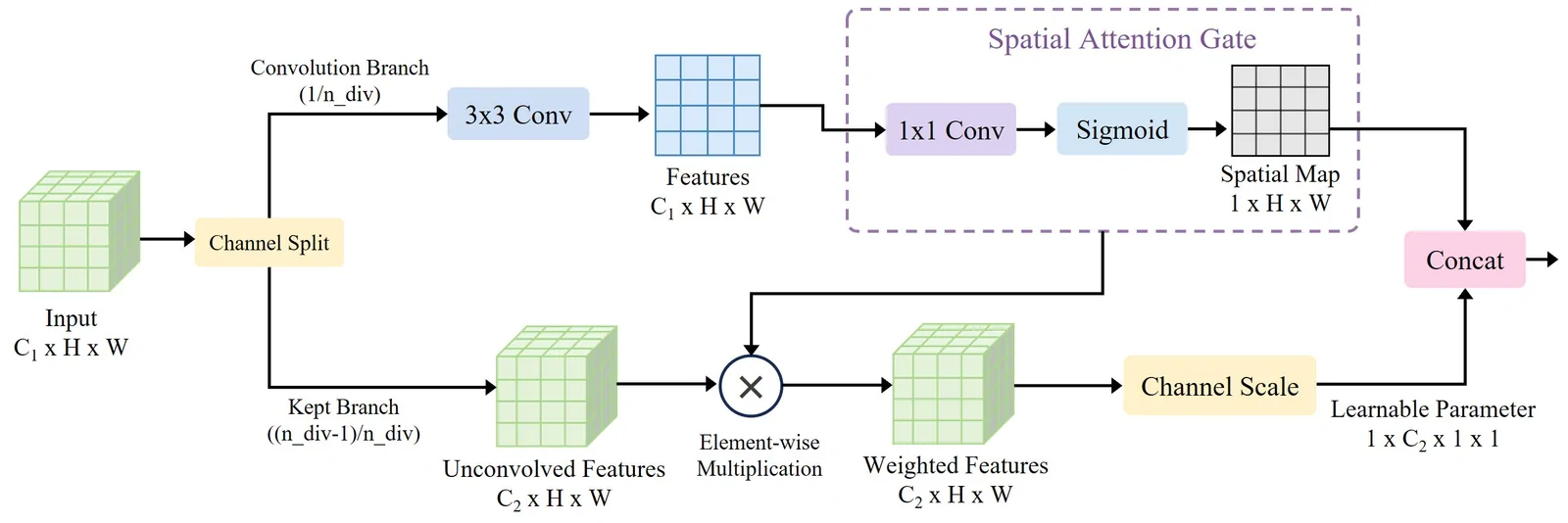
Design diagram of STGConv.

To enhance feature extraction while maintaining computational efficiency, a Spatially Weighted Token-Gated Convolution (STGConv) module was proposed. In natural greenhouse environments, shiitake mushrooms exhibit substantial variations in scale, morphology, texture, and occlusion conditions. These variations make it challenging for lightweight detectors to simultaneously preserve discriminative details and maintain low computational complexity. Unlike existing lightweight convolution methods that mainly focus on reducing redundant feature computation, STGConv introduces a spatially adaptive token gating mechanism within the partial convolution framework to achieve efficient feature selection. Specifically, the input feature channels are divided into a transformed branch and an identity branch. Instead of applying convolution uniformly to all channels, STGConv performs spatially guided feature transformation on partial channels and dynamically adjusts the contribution of informative spatial regions through a learnable gating operation. This design differs from conventional partial convolution, which uses fixed channel partitioning without considering spatial importance, and from independent attention modules that introduce additional computational branches. As shown in **Figure 7**. The proposed STGConv first divides the input feature map into a convolution branch and a preserved branch. The convolution branch extracts local contextual information via convolutional operations, while the preserved branch directly preserves the original features to reduce computational redundancy. Subsequently, a lightweight spatial attention gate generated from the convolution branch is applied to the preserved features, followed by channel-wise scaling and feature concatenation.

Given an input feature map $X\in {\mathbb{R}}^{C\times H\times W}$, STGConv first performs channel splitting, as shown in Equation 2.

(2) $$X=\left[X_{conv},X_{keep}\right]$$

where *X_conv_* and *X_keep_* denote the convolution branch and the preserved branch, respectively. Only a portion of channels participates in convolution, while the remaining channels are directly retained. This design effectively reduces redundant computation and preserves original semantic representations. The convolution branch is then utilized to generate a spatial attention map, as shown in Equation 3.

(3) $$M_{s}=\sigma \left(Conv_{1\times 1}\left(Conv_{3\times 3}\left(X_{conv}\right)\right)\right)$$

where *Conv*_3×3_(·) extracts local spatial information, *Conv*_1×1_(·) performs feature projection, and *σ*(·) denotes the sigmoid activation function. The resulting attention map *M_s_* highlights informative regions and suppresses irrelevant background interference. Finally, the spatial weights are applied to the preserved features and combined with learnable channel scaling to obtain the output feature map, as shown in Equation 4.

(4) $$Y=Concat\left(X_{conv},\gamma ⊙\left(M_{s}⊙X_{keep}\right)\right)$$

where ⊙ represents element-wise multiplication, *γ* denotes a learnable channel scaling parameter, and *Concat*(·) indicates channel concatenation. Through this process, STGConv adaptively enhances discriminative features while maintaining computational efficiency.

By coupling lightweight partial feature transformation with spatially adaptive gating, STGConv selectively enhances mushroom-related regions while suppressing irrelevant background responses. This property is particularly beneficial for multi-variety and multi-stage shiitake mushroom detection, where immature mushrooms are small and densely distributed, whereas mature mushrooms contain richer texture and larger-scale structures. Therefore, STGConv provides an efficient feature enhancement strategy that improves robustness under complex greenhouse conditions while maintaining a lightweight computational profile.

#### 2.5.2 Adaptive bilateral fusion head

**Figure 8:**
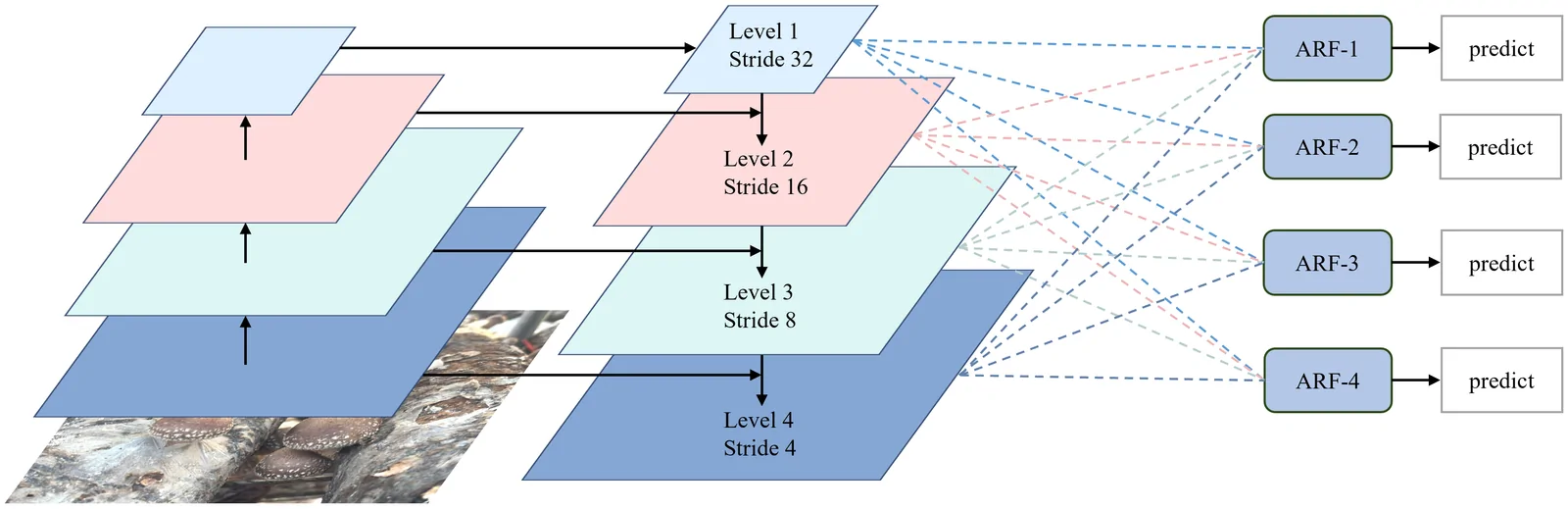
Illustration of the adaptive spatial feature fusion mechanism.

To improve the detection performance of shiitake mushrooms with diverse varieties and growth stages, an Adaptive Residual Fusion Head (ARFHead) was designed to enhance multi-scale feature representation and cross-scale information interaction. Unlike conventional adaptive feature fusion methods that mainly focus on learning scale-specific fusion weights, ARFHead introduces a residual-guided adaptive fusion strategy to simultaneously exploit complementary information from different feature levels and preserve fine-grained spatial details during feature aggregation. Based on the CSPDarkNet backbone, ARFHead adopts a four-scale detection architecture with feature maps generated at P2, P3, P4, and P5 levels, as illustrated in **Figure 8**. The high-resolution P2 and P3 features provide detailed spatial and texture information for small immature mushrooms, while P4 and P5 features provide stronger semantic representations for larger targets. Different feature levels are aligned through upsampling and downsampling operations, and adaptive weighting is employed to dynamically adjust the contribution of multi-scale features during fusion.

Different from existing adaptive fusion approaches that directly aggregate weighted features, ARFHead further incorporates residual feature propagation to retain informative spatial structures and alleviate the loss of fine-grained details caused by multi-scale transformation. This residual-guided fusion mechanism facilitates effective information exchange between shallow and deep features while maintaining computational efficiency. For shiitake mushroom detection under complex greenhouse environments, where targets exhibit significant variations in size, morphology, texture, and occlusion conditions, ARFHead enhances the representation capability of both small densely distributed mushrooms and large mature mushrooms. By combining adaptive scale selection with residual feature preservation, ARFHead improves cross-scale feature interaction and provides more discriminative feature representations for multi-variety and multi-stage shiitake mushroom classification and grading.

ARFHead employs an adaptive weighting mechanism to fuse features across four detection heads. Let $x_{i,j}^{l\leftarrow m}$ denote the feature vector at position (*i,j*) on the feature map adjusted from level *m* to level *l*. Feature fusion at the corresponding level *l* is performed as follows, as shown in Equation 5:

(5) $$y_{ij}^{l}=\alpha_{ij}^{l}\cdot x_{ij}^{1\to l}+\beta_{ij}^{l}\cdot x_{ij}^{2\to l}+\gamma_{ij}^{l}\cdot x_{ij}^{3\to l}+\delta_{ij}^{l}\cdot x_{ij}^{4\to l}$$

Where $y_{ij}^{l}$ indicates the (*i*, *j*)-th element of the output feature map *y^l^* across channels. The coefficients $\alpha_{ij}^{l}$, $\beta_{ij}^{l}$, $\gamma_{ij}^{l}$ and $\delta_{ij}^{l}$ correspond to spatial importance weights from four different levels relative to level *l*, and are adaptively determined by the network. These factors can be regarded as simple scalars shared among all channels. Enforce that: $\alpha_{ij}^{l}$ + $\beta_{ij}^{l}$ + $\gamma_{ij}^{l}$ + $\delta_{ij}^{l}$ = 1 and $\alpha_{ij}^{l}$, $\beta_{ij}^{l}$, $\gamma_{ij}^{l}$, $\delta_{ij}^{l}$ ∈ [0, 1]; and defined as shown in Equation 6:

(6) $$\alpha_{ij}^{l}=\frac{e^{\lambda_{\alpha_{ij}}^{l}}}{e^{\lambda_{\alpha_{ij}}^{l}}+e^{\lambda_{\beta_{ij}}^{l}}+e^{\lambda_{\gamma_{ij}}^{l}}+e^{\lambda_{\delta_{ij}}^{l}}}$$

Here $\;\alpha_{ij}^{l}$, $\beta_{ij}^{l}$, $\gamma_{ij}^{l}$ and $\delta_{ij}^{l}$ are defined using the softmax function, with $\lambda_{\alpha_{ij}}^{l}$, $\lambda_{\beta_{ij}}^{l}$, $\lambda_{\gamma_{ij}}^{l}$ and $\lambda_{\delta_{ij}}^{l}$ serving as control parameters respectively. The weight scalar mappings $\alpha_{ij}^{l}$, $\beta_{ij}^{l}$, $\gamma_{ij}^{l}$ and $\delta_{ij}^{l}$ are computed from the input features using 1×1 convolutional layers, enabling their learning through standard backpropagation.

#### 2.5.3 DySample

**Figure 9:**
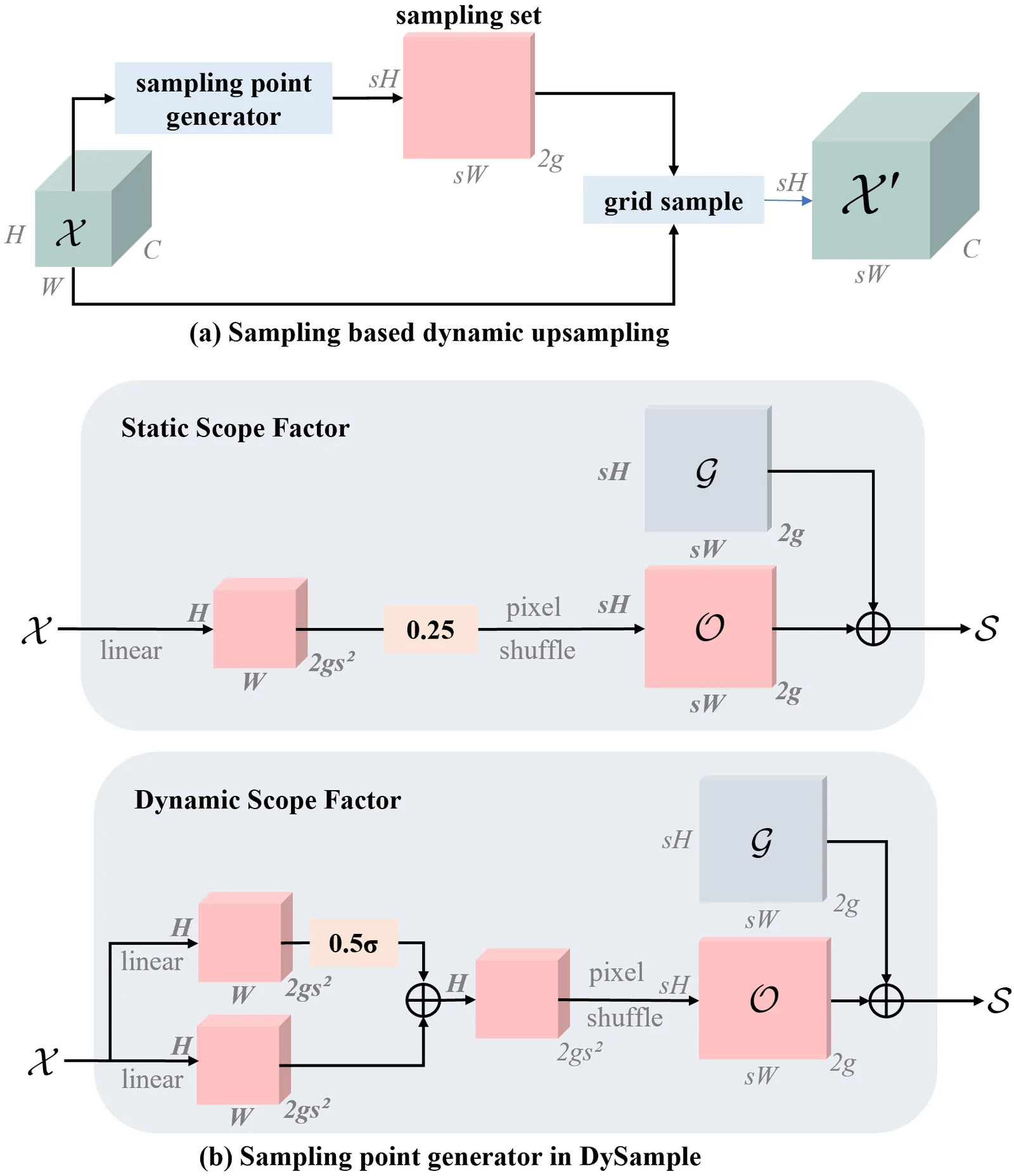
Dynamic upsampling and module design based on sampling in dysample. Input features, upscaled features, generated offsets, and original grid are denoted by X, X′, O and G respectively.

Small feature maps are often unable to capture sufficient spatial details, which can limit detection accuracy for small or densely packed objects. To address this, this study introduces the lightweight and efficient Dysample upscaling module proposed by (Liu et al., 2023) in 2023. DySample avoids complex dynamic convolution operations, significantly reducing computational burden and inference time. It performs upsampling through point sampling rather than traditional kernel-based methods, which simplifies the process and improves efficiency. Compared to conventional approaches, DySample requires fewer parameters and consumes less GPU memory, reducing overall computational cost while maintaining high-quality feature reconstruction. For the multi-varietal shiitake mushroom detection task in natural growth environments, DySample is particularly suitable because the mushrooms often appear in small sizes, irregular shapes, and densely clustered patterns. By effectively recovering fine-grained spatial details from low-resolution feature maps, DySample enhances the network’s ability to accurately locate and classify small, overlapping, or deformed mushrooms. **Figure 9** illustrates the sample-based dynamic upscaling and modular design within Dysample.

**Figure 9** illustrates how a sample set S is constructed from input features X through a sampling point generator, then resampling the input features with the grid sample function to obtain the upsampled features X′. **Figure 9** further presents two strategies for sampling point generation: static and dynamic range factors. In the static case, the offset O is produced by combining a linear layer with pixel shuffle operations under a fixed range factor, and then added to the original grid G to form S. For the dynamic case, a range factor is first estimated and then applied to adjust the offset O. Here, *σ* denotes the Sigmoid function used to generate this factor. Through these procedures, DySample aggregates contextual cues from broad receptive fields and adaptively produces kernels, thereby improving its ability to capture details of occluded targets. Importantly, DySample introduces only minor computational cost and can be seamlessly incorporated into modern network designs.

### 2.6 Experimental design

#### 2.6.1 Experimental environment

The software and hardware environment and parameters used in this experiment are shown in **Table 1**.

**Table 1:** Software and hardware parameters.

Accessories	Model
Operating system	Ubuntu 20.04.6 LTS
CPU	Intel (R) Core (TM) i7-13700K
RAM	32 GB
GRAM	24 GB
GPU	NVIDIA GeForce RTX 4090
Development environments	Python 3.9.18, torch 2.0.1 + cu117

#### 2.6.2 Experimental parameter settings

The model was trained using the SGD optimizer with a learning rate 0.01 and a weight decay of 0.005. Momentum was initially set to 0.8 for the first three warm-up epochs and then increased to 0.937. The process ran for 400 epochs with a batch size of 8. A total of 3150 original annotated images were divided into training, validation, and test sets at a ratio of 8:1:1. To ensure the fairness of the comparison, all models were retrained using the same dataset partition and identical training settings.

#### 2.6.3 Model evaluation methods

To accurately evaluate model performance, the evaluation metrics used in this study are precision, recall, F1 score, average precision (AP), and mean average precision (mAP). To evaluate real-time detection accuracy for multi-variety shiitake mushroom classification and grading, the IoU threshold for mAP evaluation was set at 0.5. The calculation formulas are presented in Equations 7–9:

(7) $$F_{1}=\frac{2\times \text{Precision}\times \text{Recall}}{\text{Precision}+\text{Recall}}$$

(8) $$mAP=\frac{1}{N}\sum_{i=1}^{N}AP_{i}$$

(9) $$AP=\int_{0}^{1}P\left(R\right)\;dR$$

FLOPs and Params are employed to measure model complexity, thereby quantifying and enhancing the evaluation of model lightweighting. The calculation formulas for the two evaluation metrics are shown in Equations 10 and 11:

(10) $$\text{FLOPs}=K^{2}\times C_{\text{in}}\times C_{\text{out}}\times H_{\text{out}}\times W_{\text{out}}$$

(11) $$\text{Params}=K^{2}\times C_{\text{in}}\times C_{\text{out}}+B$$

Where *K* represents the convolution kernel size, *C*_in_ and *C*_out_ are the numbers of channels for input and output, *H*_out_ and *W*_out_ denote the height and width of the resulting feature map, and *B* stands for the bias term.

## 3 Results and analysis

### 3.1 Ablation experiments

**Figure 10:**
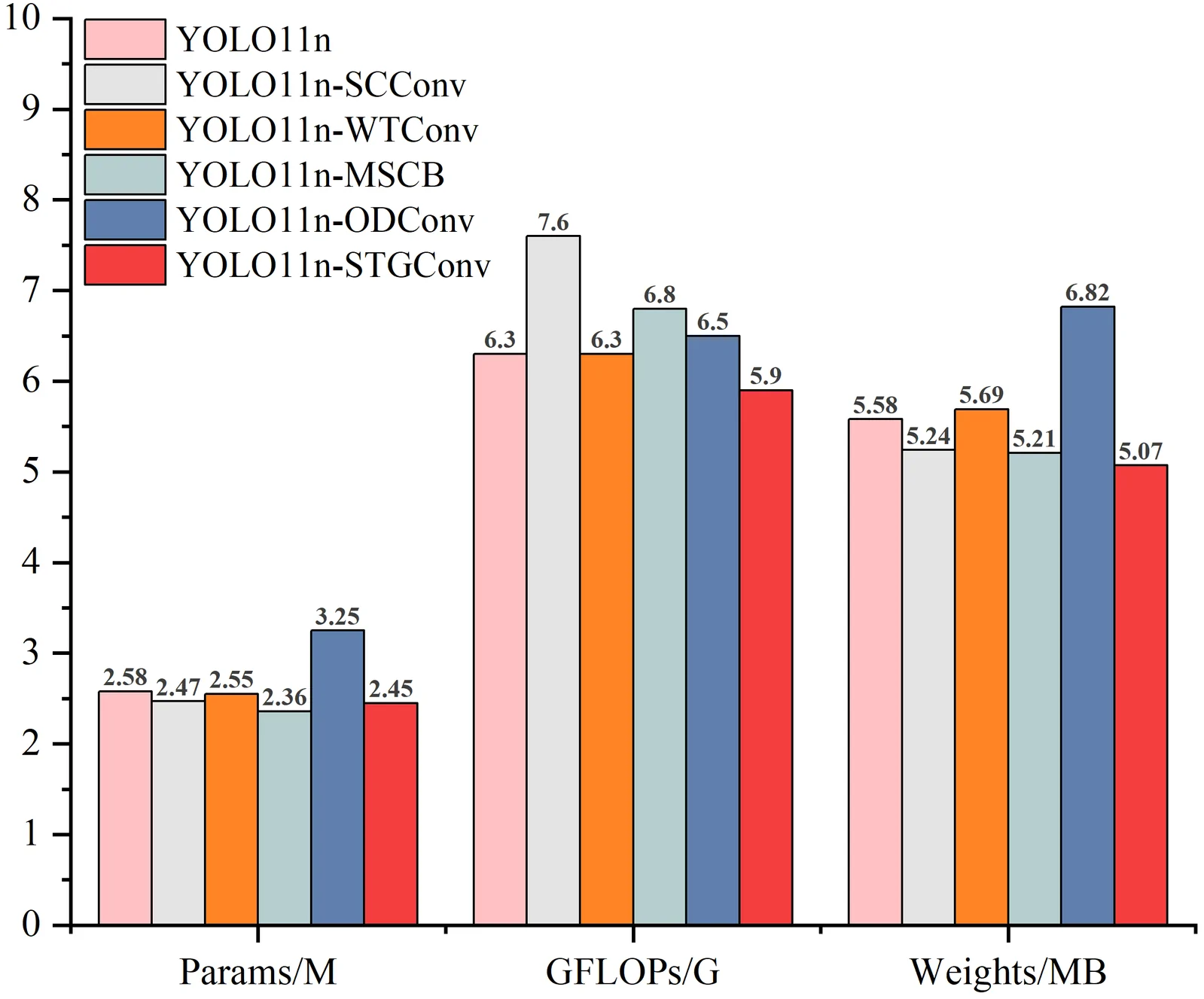
Comparison of parameters, GFLOPs, and Weights of 5 lightweight improved models with the baseline model.

To demonstrate the effectiveness of the proposed STGConv module, its performance was compared with four representative lightweight convolution operators, including SCConv (Li et al., 2023), WTConv (Finder et al., 2024), MSCB (Rahman et al., 2024), and ODConv (Li et al., 2022). As shown in **Figure 10** Among these five models, YOLO11n-STGConv exhibits significant advantages in terms of parameters and GFLOPs, reducing the parameter count to 2.45M and the computational complexity to 6.8G. STGConv reduces redundant computations and memory access within the model, enabling more efficient spatial feature extraction and significantly lowering model complexity. Compared to the baseline YOLO11n, YOLO11n-STGConv achieves a 5% reduction in parameters and a 6.85% reduction in GFLOPs, as shown in **Table 2**, while maintaining comparable accuracy.

**Table 2:** Performance comparison of YOLO11n replacing different convolution modules.

Model	Params (M)	GFLOPs (G)	Weights (MB)	mAP@0.5 (%)
YOLO11n	2.58	7.3	5.24	93.1
YOLO11n-SCConv	2.48	7.6	5.18	91.8
YOLO11n-WTConv	2.55	6.3	5.22	92.5
YOLO11n-MSCB	2.36	6.8	5.13	91.3
YOLO11n-ODConv	3.25	7.8	6.41	92.7
**YOLO11n-STGConv**	**2.45**	**6.8**	**5.19**	**92.9**

Bold values indicate the results obtained by the proposed model.

**Figure 11:**
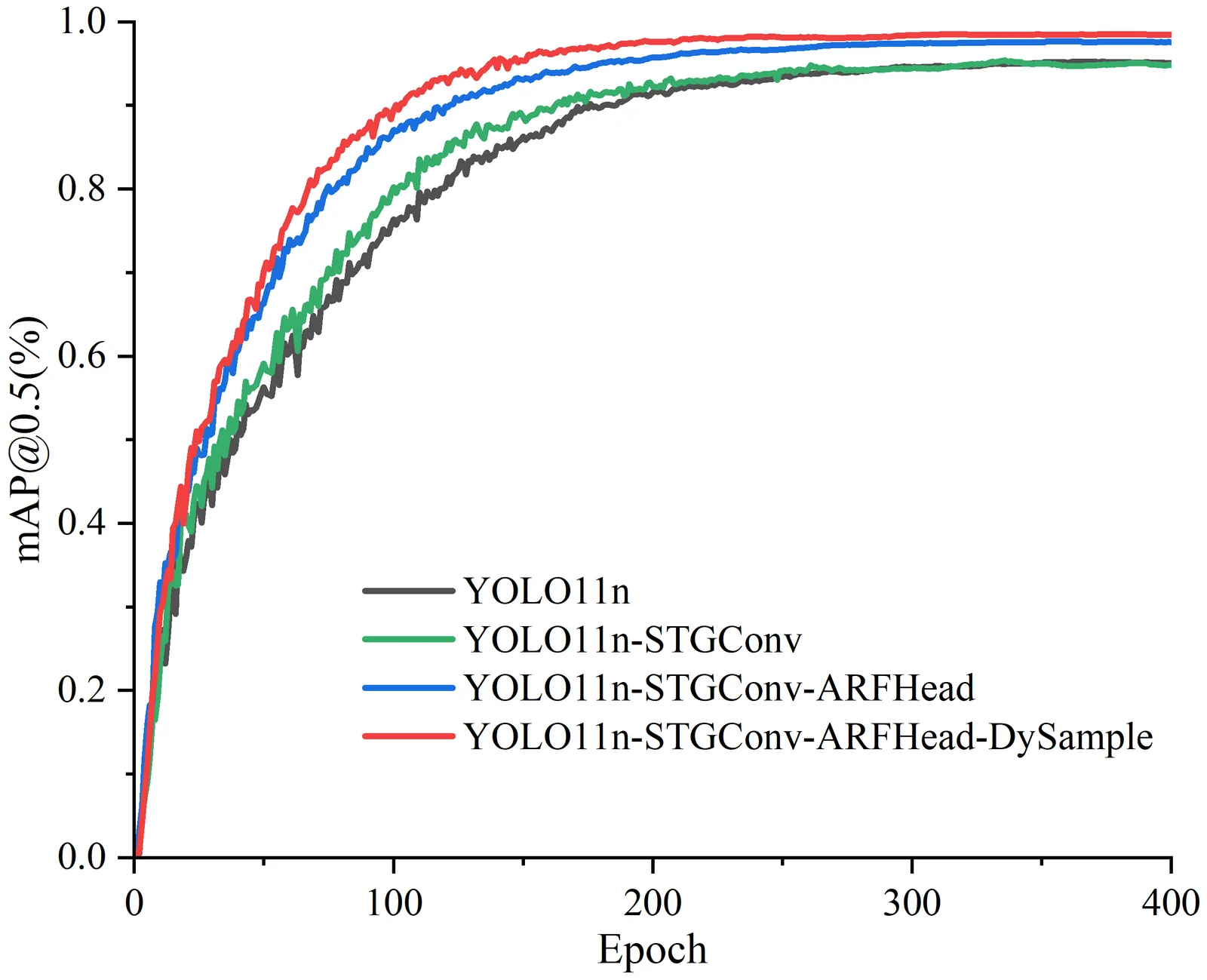
Comparison of mAP@0.5 values of ablation experiments.

To evaluate the contribution of each component, ablation experiments were conducted on the proposed YOLO-RTSCGD, as shown in **Table 3** and **Figure 11**. Replacing the standard convolution with STGConv reduced the parameter count and computational cost from 2.58 M and 7.3 GFLOPs to 2.45 M and 6.8 GFLOPs, respectively, while resulting in a slight decrease in mAP@0.5 from 93.1% to 92.9%. The introduction of ARFHead increased mAP@0.5 to 96.2%, indicating that enhanced multi-scale feature fusion improves detection performance. DySample also improved the detection accuracy, achieving amAP@0.5 of 94.3%, suggesting that preserving feature details during upsampling is beneficial for feature representation. When combined with STGConv, ARFHead and DySample increased mAP@0.5 to 97.3% and 95.3%, respectively. Furthermore, the combination of ARFHead and DySample achieved a mAP@0.5 of 96.7%. Integrating all three modules yielded the highest mAP@0.5 of 97.9%, while maintaining 3.92 M parameters and 10.6 GFLOPs. These results indicate that STGConv contributes to model lightweighting.

**Table 3:** Results of the ablation experiment.

Basic model	+STGConv	+ARFHead	+DySample	Params (M)	GFLOPs (G)	Weights (M)	mAP@0.5 (%)	mAP@0.5:0.95 (%)
✓				2.58	7.3	5.24	93.1	77.8
✓	✓			2.45	6.8	5.19	92.9	77.5
✓		✓		3.96	12.3	8.61	96.2	84.1
✓			✓	2.56	7.4	5.22	94.3	80.9
✓	✓	✓		3.94	11.2	8.41	97.3	85.8
✓	✓		✓	2.55	7.1	5.21	95.3	82.5
✓		✓	✓	3.95	11.7	8.53	96.7	84.9
✓	✓	✓	✓	**3.92**	**10.6**	**8.27**	**97.9**	**86.7**

Bold values indicate the results obtained by the proposed model.

ARFHead enhances multi-scale feature fusion, and DySample improves feature reconstruction during upsampling, jointly contributing to the overall detection performance of YOLO-RTSCGD.

### 3.2 Comprehensive comparative analysis of model performance

To verify the superiority of the YOLO-RTSCGD model in real-time classification and grading of multiple varieties of shiitake mushrooms, we conducted a comprehensive comparative experiment with it and other mainstream object detection models. To ensure a fair comparison, all models were trained and evaluated using the same dataset, input image size, data augmentation strategies, training epochs, optimization settings, and hardware environment. We evaluated nine performance indicators: parameters, GFLOPs, weights, precision, recall, F1 score, mAP@0.5, mAP@0.5:0.95 and frames per second (FPS). The detailed results are shown in **Table 4**. The YOLO-RTSCGD model achieves the highest mAP@0.5 value, reaching 97.9%. Compared with Faster R-CNN, RT-DETR, YOLOv5n, YOLOv8n, YOLOv9t (without auxiliary branches), YOLOv10n, and YOLO11n, it achieves improvements of 4.1%, 3.6%, 6.4%, 5.3%, 8.5%, 5.4%, and 4.8%, respectively. It also demonstrated excellent performance across other evaluation metrics, including precision, recall, and F1, achieving 96%, 96.3%, and 96.2%, respectively, outperforming all other models. Regarding computational efficiency, YOLO-RTSCGD significantly reduces GFLOPs by 94% compared to Faster R-CNN, decreasing the number of parameters to 3.92M and the model size to 8.27MB.

**Table 4:** Experimental results of YOLO-RTSCGD compared with other models.

Model	Params (M)	GFLOPs (G)	Weights (M)	Precision (%)	Recall (%)	F1 (%)	mAP@0.5 (%)	mAP@0.5:0.95 (%)	FPS (f/s)
Faster R-CNN	35.41	178	278.31	93.1	88.3	90.6	93.8	78.2	26.1
RT-DETR	32.8	87.8	76.2	93.8	90	91.8	94.3	79.5	89.1
YOLOv5n	2.51	7.1	5.18	91.8	86.0	88.8	91.5	75.7	109.5
YOLOv8n	3.01	8.1	6.14	92.4	85.3	88.7	92.6	77.9	116.8
YOLOv9t	1.97	7.6	4.56	87.7	81.7	84.6	89.4	74.3	43.3
YOLOv10n	2.70	8.2	5.66	92.5	87.2	89.8	92.5	77.6	74.8
YOLO11n	2.58	7.3	5.24	93.5	87.8	91.0	93.1	77.8	86.7
**YOLO-RTSCGD**	**3.92**	**10.6**	**8.27**	**96.0**	**96.3**	**96.2**	**97.9**	**86.7**	**110.8**

Bold values indicate the results obtained by the proposed model.

**Figure 12:**
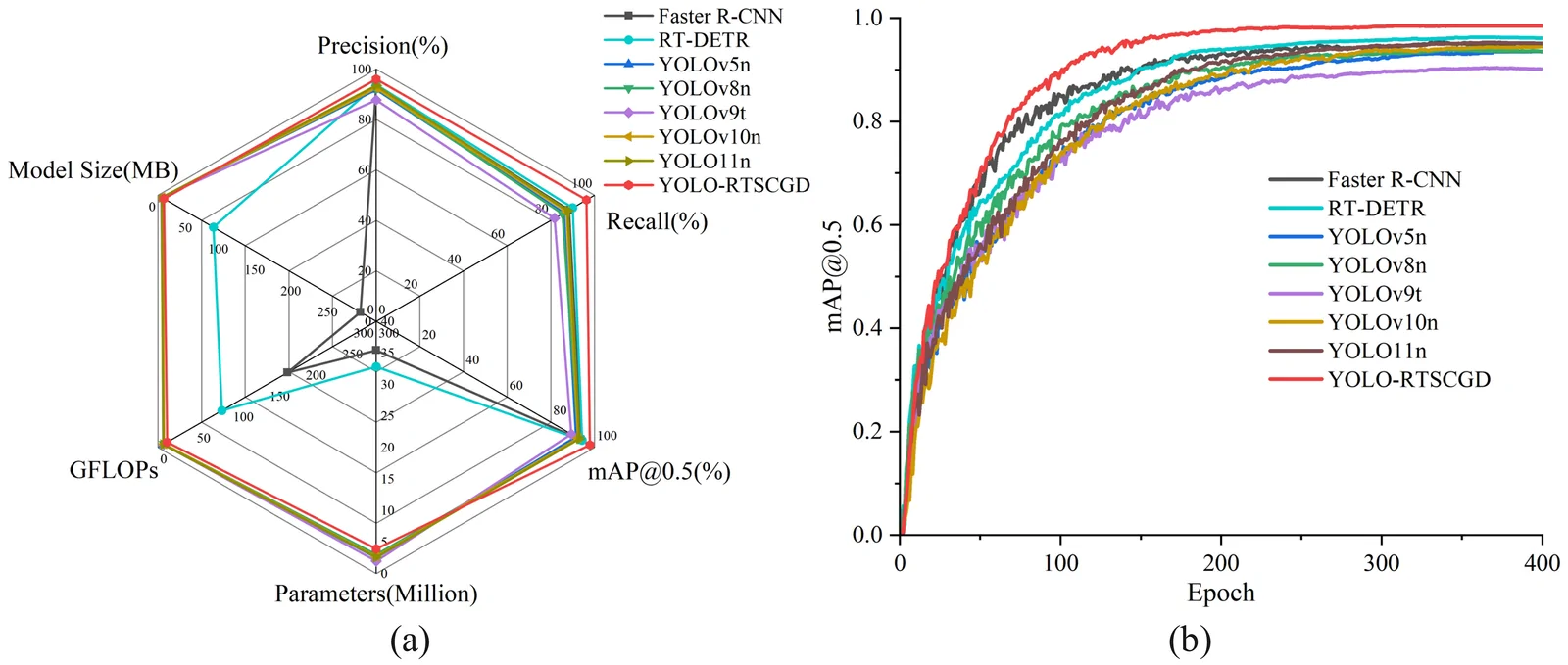
Comprehensive performance comparison of different detection models: **(a)** radar chart of overall performance across accuracy and complexity metrics (Precision, Recall, mAP@0.5, Parameters, GFLOPs, and Model Size); **(b)** mAP@0.5 convergence curves during training over 400 epochs.

The experimental results were visualised and analysed using radar charts, with the findings in **Figure 12**. The FPS evaluation underscores the model’s efficiency, achieving 110.8 frames per second on a CPU, indicating its suitability for real-time use under limited computing resources. In addition, visualizing the training process helps capture dynamic variations more effectively and reveals the improvement rate and convergence characteristics of different models. A visual comparison of the mAP@0.5 performance of different models during training was conducted, as shown in **Figure 12**. It is evident that YOLO-RTSCGD (red curve) exhibits a faster convergence rate during the initial training phase and converges earlier, indicating superior learning capabilities and a more stable optimization process.

**Figure 13:**
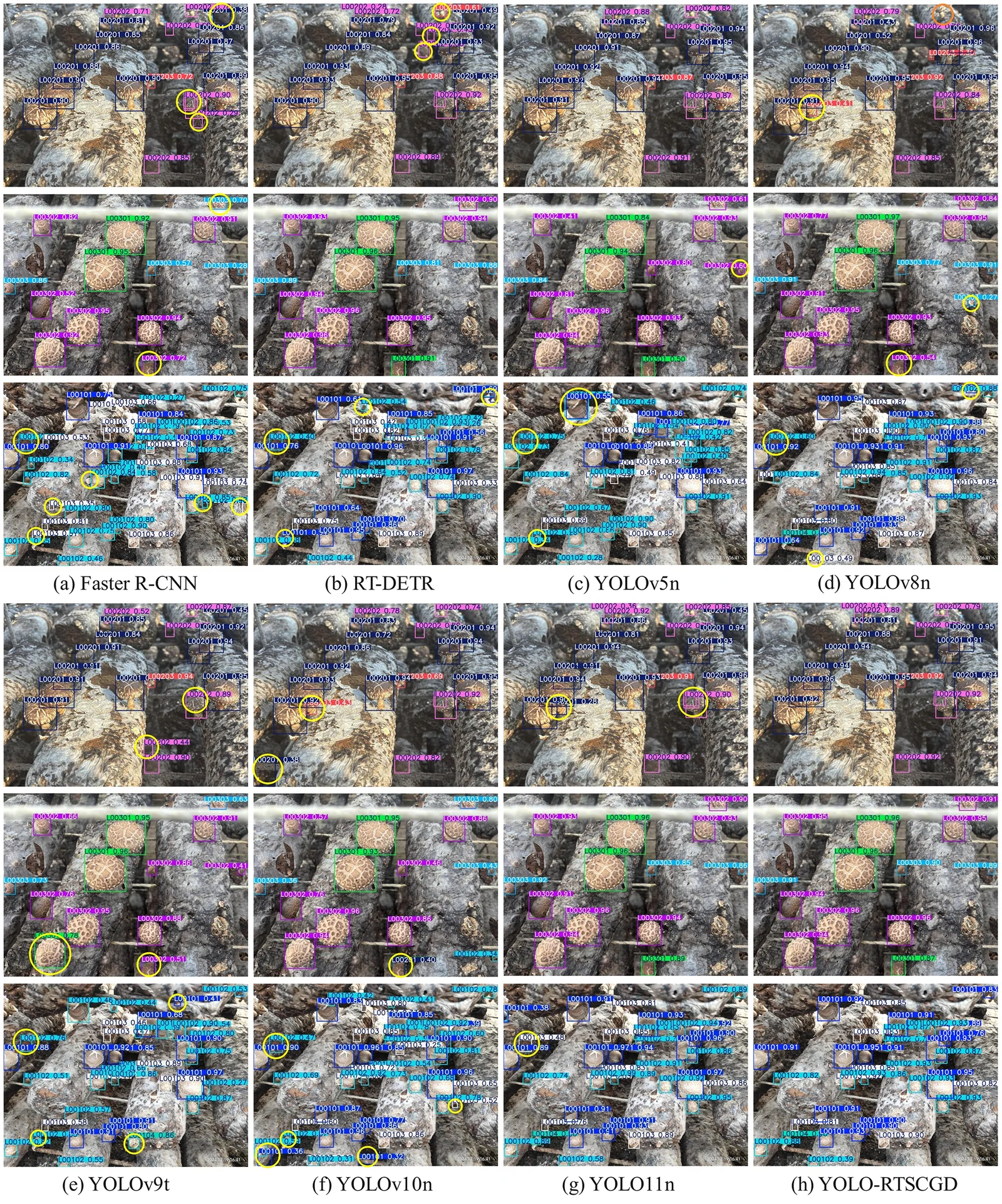
Detection results for different models: **(a)** Faster R-CNN, **(b)** RT-DETR, **(c)** YOLOv5n, **(d)** YOLOv8n, **(e)** YOLOv9t, **(f)** YOLOv10n, **(g)** YOLO11n, and **(h)** YOLO-RTSCGD. Yellow circles indicate false positives (misdetections), while orange circles indicate false negatives (missed detections).

After training different object detection models on the same dataset, real-time classification and grading inference were performed on naturally grown multi-varietal shiitake mushrooms, followed by practical detection. The comprehensive test results and performance metrics are shown in **Figure 13**. The Faster R- CNN, RT-DETR, YOLOv5n, YOLOv8n, YOLOv9t, YOLOv10n, and YOLO11n models exhibit frequent misclassification errors during real-time inference and practical testing. These problems manifest as misidentifying mushroom stems or background textures as individual shiitake mushrooms and incorrectly classifying growth stages. Furthermore, YOLOv8n shows missed detections. In contrast, the YOLO-RTSCGD model proposed herein accurately detects all varieties of shiitake mushrooms within an image, along with their respective growth stages, with no instances of misclassification or omission. It demonstrates significantly superior detection performance compared to other models, particularly in complex scenarios featuring overlapping and occluded mushrooms. Experimental results demonstrate that YOLO-RTSCGD exhibits superior robustness and generalization, enabling it to address the challenges of shiitake mushroom detection in natural growth environments. Furthermore, it maintains high detection performance and accuracy even for occluded and overlapping targets.

### 3.3 Confusion matrix analysis

**Figure 14:**
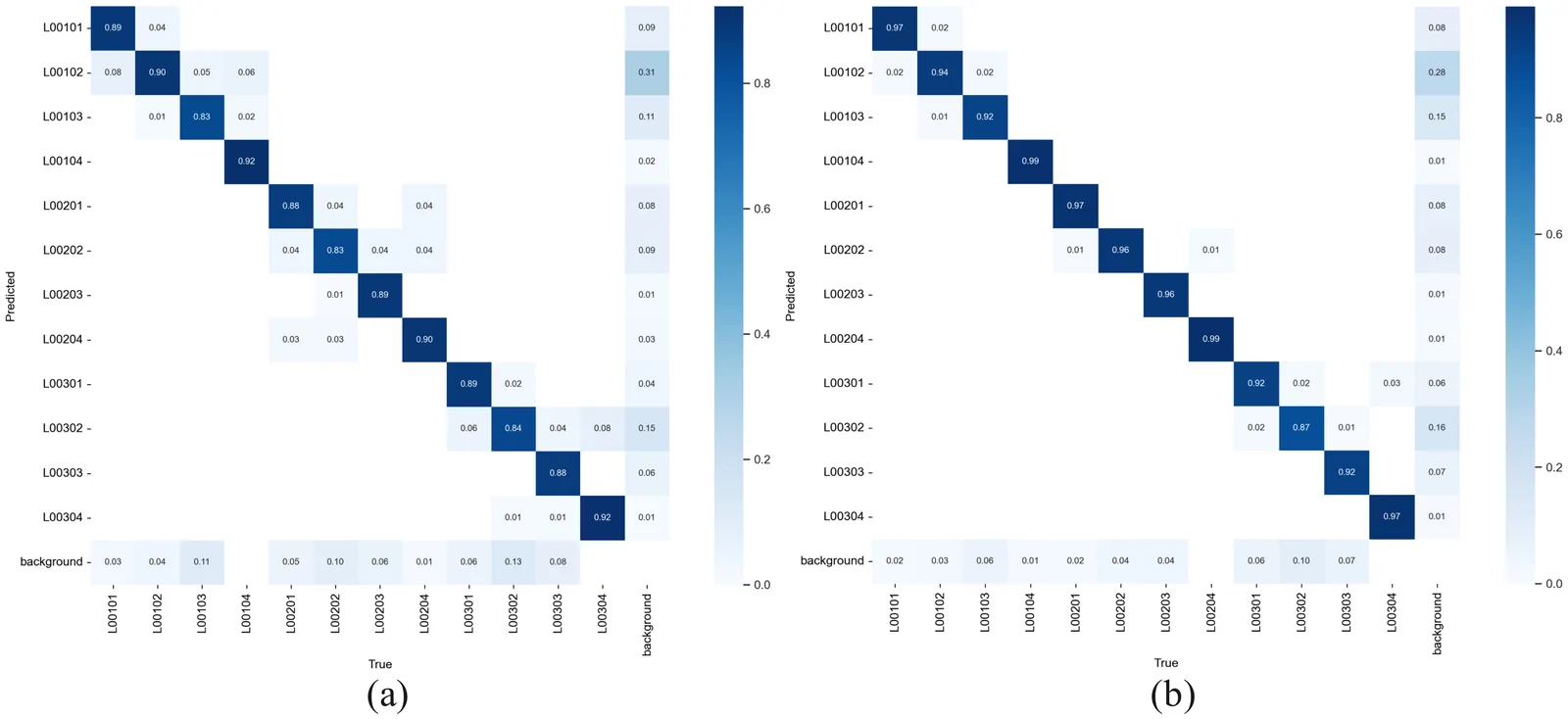
Normalized confusion matrices for multi-class detection: **(a)** YOLO11n; **(b)** YOLO-RTSCGD. The diagonal elements represent the classification accuracy for each category, while off-diagonal elements indicate classification errors and background confusion.

The confusion matrix provides a detailed view of category-wise prediction results and helps identify potential misclassification patterns that may not be fully reflected in overall evaluation metrics. Normalized confusion matrices of YOLO11n and the proposed YOLO-RTSCGD model are presented in **Figure 14**. Compared with overall metrics such as precision and mAP, confusion matrices provide a more intuitive visualization of inter-class relationships and reveal which categories are more likely to be confused. As shown in **Figure 14**, the baseline YOLO11n model exhibits several noticeable off-diagonal responses, indicating the presence of inter-class confusion. Most misclassifications occur between neighboring growth stages within the same variety, which can be attributed to their highly similar morphological characteristics, texture patterns, and color distributions. In addition, several categories show relatively high confusion with the background, suggesting that the baseline model is susceptible to missed detections under complex cultivation conditions.

In contrast, the confusion matrix of YOLO-RTSCGD is shown in **Figure 14** demonstrates stronger diagonal responses and substantially fewer off-diagonal activations. The diagonal values of most categories exceed 0.90, indicating that the proposed model can accurately distinguish different mushroom categories. Compared with YOLO11n, background confusion is noticeably reduced, implying that fewer targets are missed during detection. This improvement is particularly evident for categories that exhibited relatively high confusion in the baseline model. Although some confusion remains among several visually similar categories, the overall error distribution is significantly reduced. These results indicate that the proposed YOLO-RTSCGD model exhibits stronger feature discrimination and improved robustness in dense, complex mushroom cultivation environments.

### 3.4 Model interpretability analysis

**Figure 15:**
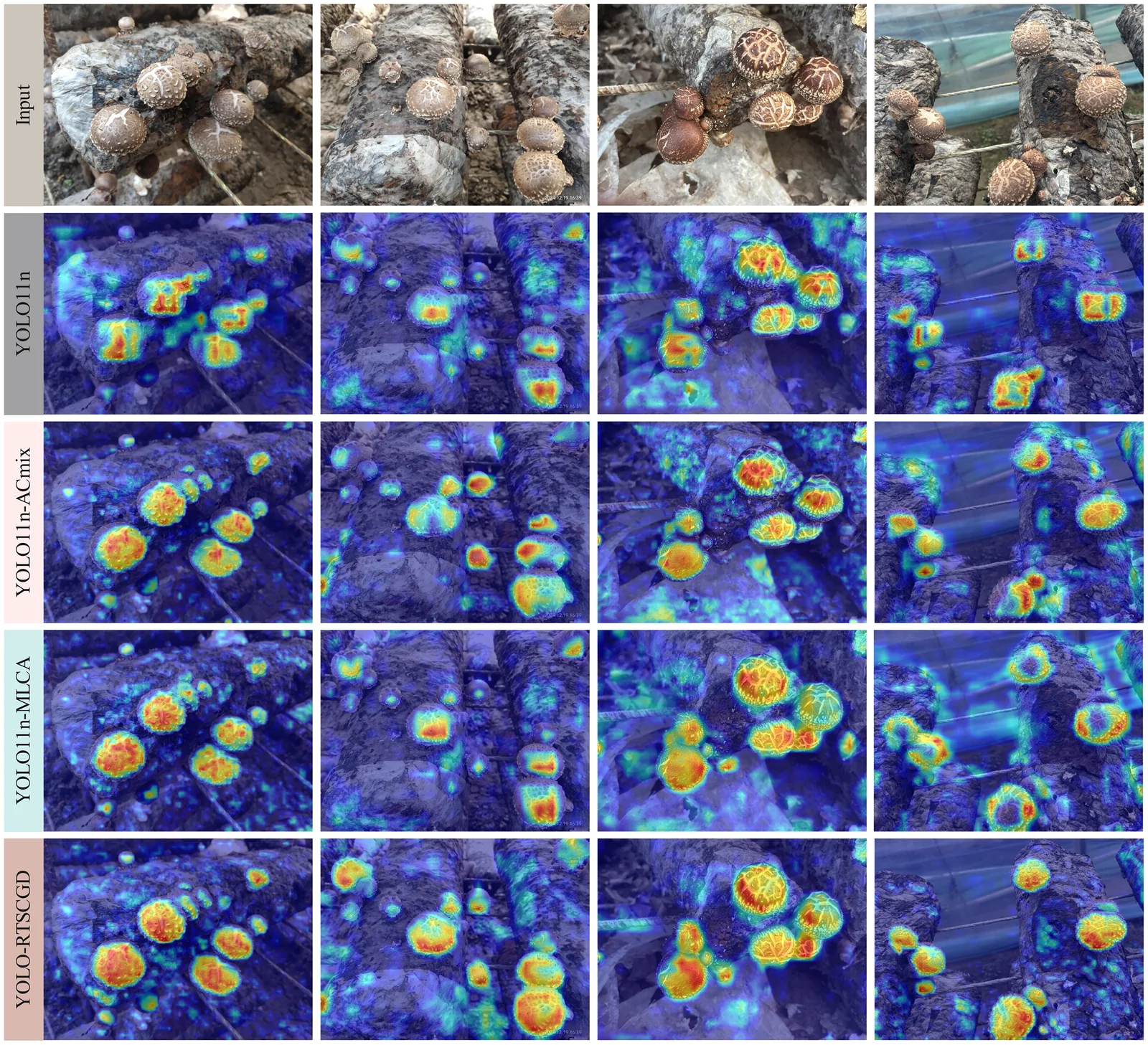
Grad-CAM output for different models.

Deep learning models can achieve high accuracy in specific detection tasks, but require improved interpretability. To better understand the learning capabilities of the models proposed in this study, this study employs the Grad-CAM (Selvaraju et al., 2016) method to visually analyze feature responses and validate the detection capabilities of YOLO11n and its improved variants. As shown in **Figure 15**, Grad-CAM generates a class activation map based on gradient information from the target category, revealing the key focus regions during the model’s detection process. Colors ranging from blue to red indicate varying degrees of attention, from low to high. Visualization results from the YOLO11n model reveal issues across multiple scenarios, including dispersed activation regions, unstable object responses, and ineffective local object attention. Two representative attention mechanism modules, ACmix (Pan et al., 2022) and MLCA (Wan et al., 2023), were introduced as comparative experiments based on the original model to further validate the proposed model’s efficacy. These two mechanisms enhance the model’s feature-focusing capability to a certain extent, boosting the response intensity in certain target regions. However, incomplete local responses persist under small object detection, boundary perception, and complex background interference. In contrast, the YOLO-RTSCGD model enhances multi-scale feature fusion by incorporating the ARFHead, which strengthens cross-scale information interaction and improves the representation of objects across different growth stages and scales. To further refine feature extraction, the STGConv module is introduced to enhance local structural modeling and contextual perception while maintaining computational efficiency. In addition, DySample is employed as an adaptive upsampling strategy to improve spatial detail preservation during feature reconstruction. This combined design produces more concentrated and continuous high-response regions across diverse image conditions, particularly in scenarios involving dense distribution, severe occlusion, and significant scale variation. Consequently, it effectively improves both detection accuracy and localization completeness.

### 3.5 Real-scene detection performance analysis

**Figure 16:**
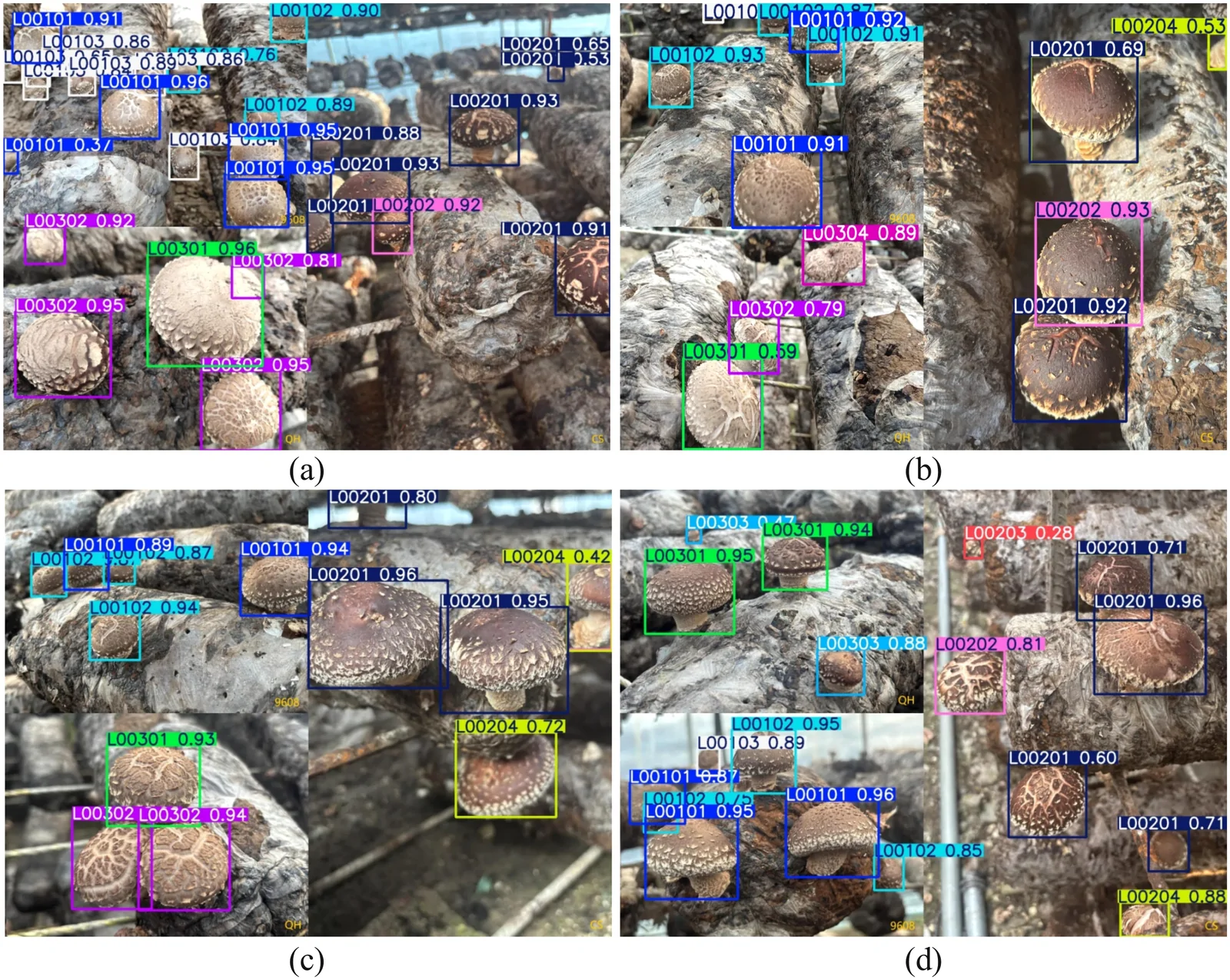
Test results across multiple scenarios: **(a)**, **(b)**, **(c)**, and **(d)** represent detection results for composite shiitake mushroom images created by stitching together images of three distinct varieties captured in different environments.

Multi-scenario detection performance analysis experiments were conducted to further validate the performance of the shiitake mushroom detection model in real-world complex environments. We collected a new, independent inference dataset from different shiitake mushroom cultivation greenhouses. The images were acquired under diverse lighting conditions, planting layers, mushroom density distributions, and viewing perspectives to better reflect practical cultivation environments. This dataset was completely independent of the training and validation datasets and was not involved in model training, validation, or parameter tuning. By combining and proportionally stitching images of three different shiitake mushroom varieties, we generated 210 complete images containing the mixed varieties. These images simulated the detection task of mixed varieties in real scenarios. The experimental results are shown in **Figure 16**. To ensure the validity of test results, each stitched image is labeled in the lower right corner with information about the corresponding shiitake mushroom variety. Specifically, “9608” denotes the first variety, “Chunsheng No. 1” is designated as ‘CS’ for the second variety, and “Qihe No. 9” is defined as ‘QH’ for the third variety. Test results demonstrate that when multiple varieties of shiitake mushrooms appear together in the same image, YOLO-RTSCGD can still accurately distinguish each variety and precisely identify different growth stage categories within them. The average detection time per image is approximately 20 milliseconds, with individual targets detected and identified at microsecond-level latency. This result demonstrates the model’s high detection accuracy and speed and exhibits outstanding stability and practicality in diverse environments. It provides robust technical support for its promotion and application in actual shiitake mushroom cultivation and sorting scenarios.

### 3.6 Applicability validation of YOLO-RTSCGD

**Figure 17:**
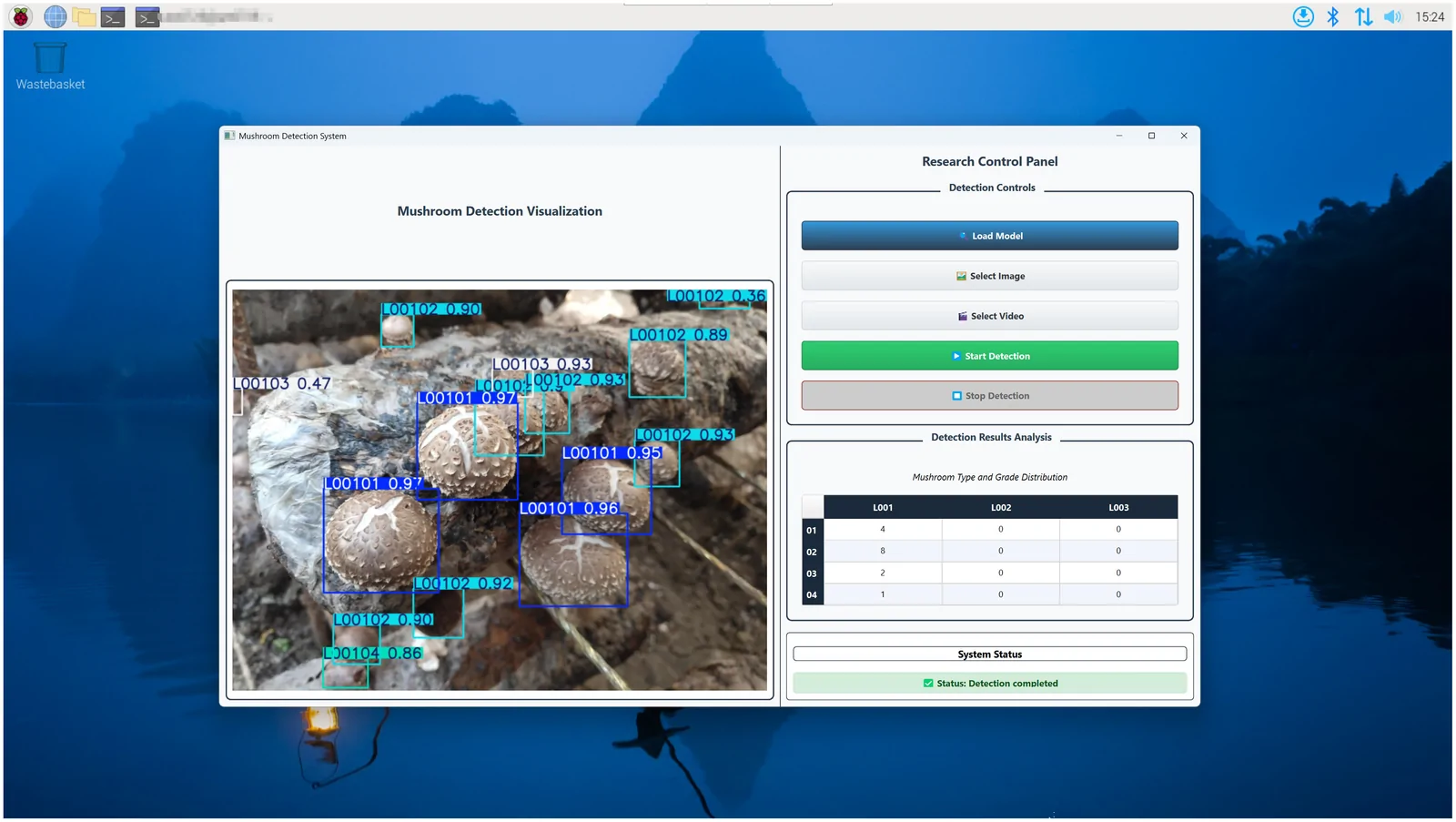
Results image from loading the YOLO-RTSCGD model on a Raspberry Pi using the shiitake mushroom detection system developed with PySide6.

We integrated the trained YOLO-RTSCGD model into computer software to rapidly identify and classify mushroom targets. We constructed and deployed a mushroom detection system based on a Raspberry Pi. This system utilizes the Raspberry Pi 4B as an edge computing platform, combined with the ONNX Runtime inference engine to achieve efficient deployment and operation of the YOLO-RTSCGD model. The system interface uses the PySide6 framework, providing a cross-platform graphical user interface. It includes functional modules for model loading, image/video import, detection execution, result visualization, and statistical analysis. The overall system architecture is shown in **Figure 17**. After completing the detection task, the system displays real-time visualizations of detection boxes, confidence scores, and category labels for shiitake mushrooms within the image. It simultaneously performs statistical counting for different mushroom categories and aggregates results by growth stage, facilitating subsequent management decisions and yield assessments. After extensive experimentation and prolonged trial operation, this system demonstrates rapid and accurate detection, stable performance, real-time responsiveness with no latency issues, a user-friendly interface, and straightforward deployment. It imposes minimal requirements on terminal configurations, enabling streamlined deployment on low-power desktop and mobile smart devices. The system achieves real-time, precise identification and classification of shiitake mushroom targets, offering significant practical value.

**Figure 18:**
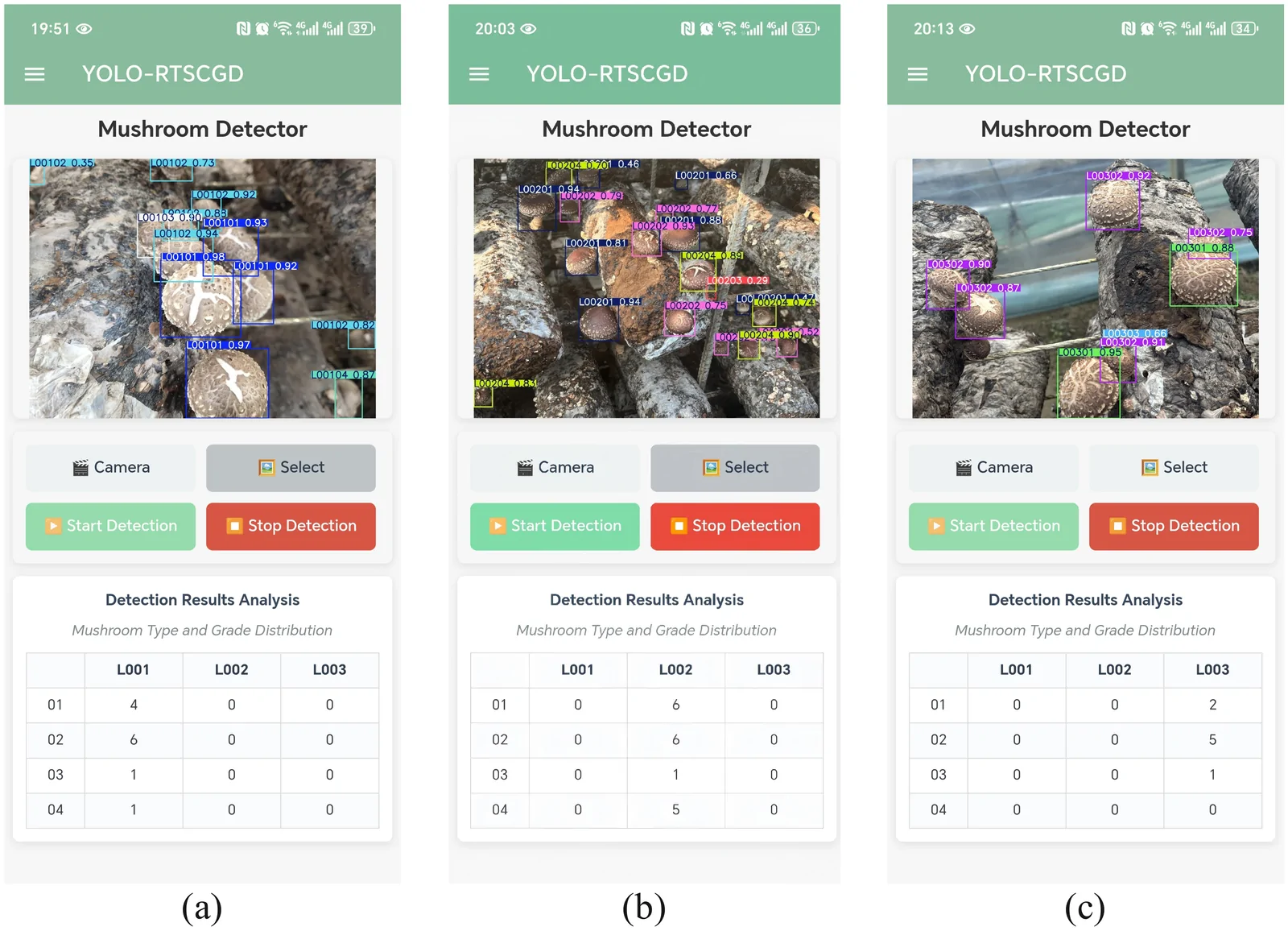
Android application developed for real-time detection and grading of multiple varieties of shiitake mushrooms. **(a)** Detection of the 9608 variety. **(b)** Detection of the Chunsheng No. 1 variety. **(c)** Detection of the Qihe No. 9 variety.

To evaluate the practical performance of the YOLO-RTSCGD model on mobile devices and validate its deployment feasibility in mobile or field environments, a mobile application integrating real-time detection capabilities for classifying and grading multiple varieties of shiitake mushrooms was developed using Android Studio. This application successfully ran on the target Android device. For this purpose, the Honor Magic6 Android smartphone was selected as the experimental platform to optimize the model for mobile deployment. Given the limited computational power of mobile devices, the model is initially transformed from the original ‘pt’ format into ONNX, with quantization applied in this step to effectively lower memory consumption and computational demands. The quantized ONNX model is then further converted into the “bin” and “param” files required by NCNN, an open-source, lightweight inference framework designed for efficient deployment on mobile platforms. It enables lightweight deployment without relying on third-party libraries and is suitable for various edge computing scenarios. Experiments demonstrate that this application can perform real-time detection of shiitake mushroom targets in images under natural growth conditions, producing visual outputs including category labels, confidence scores, and detection bounding boxes. It also features automatic counting of different categories of shiitake mushrooms and classification of their growth stages, facilitating subsequent yield assessment and quality analysis. **Figure 18** shows the application results of real-time detection for the classification and grading of three types of shiitake mushrooms in actual environments.

## 4 Discussion

This study presents a lightweight real-time framework for classification and grading of multi-varietal shiitake mushrooms under natural cultivation conditions. The proposed method achieved a favourable balance between detection accuracy and computational efficiency, demonstrating its suitability for practical applications in edible fungi production. The random combination strategy of multiple data augmentation methods improved model robustness under complex backgrounds and varying mushroom appearances, highlighting the importance of data diversity for reliable detection in natural environments. The proposed model also demonstrated strong deployment performance on resource-constrained devices via ONNX-based inference, enabling its application in real-time monitoring scenarios. Although the current dataset contains three representative shiitake mushroom varieties across 12 categories, the lightweight architecture provides flexibility for adapting to additional varieties through transfer learning or fine-tuning with limited annotated samples.

However, the current framework still has certain limitations. The dataset mainly covers three representative shiitake mushroom varieties, and its generalisation capability to a wider range of varieties requires further validation. In addition, although the proposed model showed robust performance under natural cultivation conditions, its performance under more diverse scenarios, such as extreme illumination variations, severe occlusion, and complex cultivation environments, needs further investigation. Future studies will focus on cross-variety generalisation, few-shot learning strategies, and large-scale field validation to further improve model scalability. Beyond algorithm development, the proposed framework provides a basis for integration into intelligent cultivation systems. By combining real-time detection with image acquisition platforms, the method has potential applications in growth monitoring, harvest-ready mushroom identification, and yield estimation. Further work will focus on system-level integration and validation under more diverse cultivation environments.

## 5 Conclusion

This paper addresses the need for real-time detection and grading of multi-varietal shiitake mushrooms in their natural growth state by proposing a YOLO-RTSCGD intelligent detection method and system. The main conclusions of the study are as follows:

1. By collecting large amounts of image data of three representative mushroom varieties on site, we completed the fine-grained annotation of 12 categories of targets based on the maturity and morphological grading standards commonly used in actual production and constructed a dataset with a complete structure and precise semantics.
2. This study proposed the YOLO-RTSCGD model based on detecting real growing mushroom targets. The model integrates multiple innovations, including STGConv, ARFHead, and dynamic upsampling DySample, which together enhance feature extraction, improve fusion efficiency, enhance the model’s focus on the structural details of mushrooms, and enhance the model’s generalisation ability for specific targets of growing mushrooms in real complex environments. It can improve detection effects while maintaining efficiency.
3. The experimental results indicate that YOLO-RTSCGD surpasses other object detection models, reaching 97.9% mAP@0.5, 96% precision, 96.3% recall, and a 96.2% F1 score. With only 3.92M parameters and a size of 8.27MB, it delivers an inference speed of 110.8 FPS, meeting the accuracy and efficiency demands of resource-limited scenarios. Moreover, the model has been deployed on both desktop and Android platforms, demonstrating an effective balance between detection accuracy and computational efficiency, and supporting practical real-time applications on edge devices.
4. Although this study has achieved specific results in data construction, model design, and system integration, some shortcomings remain. The system does not yet support detection scenarios involving more varieties or extreme conditions. Future efforts should focus on expanding dataset diversity and collection conditions. Additionally, enhancing model integration and deployment on edge devices will facilitate large-scale adoption in precision agriculture.

## Data Availability

The raw data supporting the conclusions of this article will be made available by the authors, without undue reservation.
